# Knowledge Graph and Large Language Model-Based Analysis of fMRI Brain Functional Neuroimaging Research

**DOI:** 10.3390/bioengineering13080945

**Published:** 2026-08-21

**Authors:** Zhenni Liu, Hanzhen Ouyang, Xuanzi Liu, Huajuan Mao, Weihui Dai, Yan Kang

**Affiliations:** 1 School of Management, Fudan University, Shanghai 200433, China; 25110690015@m.fudan.edu.cn (Z.L.); ouyanghanzhen@fudan.edu.cn (H.O.); 2 School of Pharmacy, Guizhou University, Guiyang 550025, China; cp.xzliu25@gzu.edu.cn; 3 The First Affiliated Hospital of Naval Medical University, Shanghai 200433, China; 4 School of Humanities and Management Sciences, Southwest Medical University, Luzhou 646000, China

**Keywords:** knowledge graph, large language model, multimodal neuroimaging, functional magnetic resonance imaging (fMRI), biomarker

## Abstract

The rapid growth of multimodal neuroimaging research has produced fragmented literature that limits systematic characterization of cross-modal relationships and disease-specific knowledge structures. To address this, we constructed a multimodal neuroimaging knowledge graph from 1838 peer-reviewed studies (2016–2026) spanning fMRI, EEG, fNIRS, and PET, using an LLM-based extraction and retrieval-augmented semantic merging pipeline. The resulting graph comprised 4190 nodes and 7007 edges, exhibiting a scale-free topology with a dominant connected component covering 76.6% of nodes. Alzheimer’s disease, the hippocampus, and fMRI/PET emerged as the most central hubs linking disease, anatomical, and methodological dimensions. Louvain community detection identified 25 functional modules, with seven major communities—centered on Alzheimer’s biomarker integration, molecular/fluid imaging, and psychiatric functional connectivity—forming the field’s core structure. Cross-modal analysis revealed the strongest coupling between fMRI and PET, indicating high methodological convergence. At the disease level, Alzheimer’s disease displayed a mature, hierarchically organized biomarker system, whereas major depressive disorder and chronic pain showed diffuse, less consolidated knowledge structures. These results reveal pronounced disparities across neuroimaging research domains and demonstrate that LLM-augmented knowledge graphs can systematically uncover latent structural organization relevant to multimodal integration and biomarker discovery.

## 1. Introduction

The rapid development of brain imaging techniques has provided essential tools for investigating human cognitive neural mechanisms and the pathological basis of brain disorders. Functional magnetic resonance imaging (fMRI), with millimeter-level spatial resolution, enables precise localization of task-related brain activation and has become a cornerstone for functional brain mapping [1]. Electroencephalography (EEG), characterized by millisecond temporal resolution, captures fast neural dynamics through synchronized electrical activity of neuronal populations [2]. Functional near-infrared spectroscopy (fNIRS) measures cortical hemodynamic responses through changes in blood oxygenation and is particularly suited for naturalistic and movement-related paradigms [3]. Positron emission tomography (PET) provides molecular-level insights into brain metabolism and neurotransmitter distribution [4]. These modalities offer complementary perspectives on brain function, spanning hemodynamic, electrophysiological, optical, and molecular dimensions. However, due to fundamental differences in imaging principles and signal characteristics, each modality captures only a partial view of brain activity, which makes it difficult for single-modality approaches to fully characterize the complexity of large-scale brain networks.

This limitation is particularly evident in major brain disorders such as major depressive disorder, Alzheimer’s disease, and schizophrenia, which involve distributed abnormalities across multiple brain regions, temporal scales, and neurophysiological processes, reflecting highly complex and heterogeneous pathological mechanisms. For instance, functional magnetic resonance imaging (fMRI) has identified disrupted functional connectivity between the anterior cingulate cortex and the amygdala in patients with major depressive disorder, yet it cannot capture millisecond-scale rapid neuroelectrical dynamics, such as frontal alpha asymmetry, in the way that electroencephalography (EEG) does [5,6]. Functional near-infrared spectroscopy (fNIRS), while capable of monitoring real-time prefrontal cortical oxygenation changes [7], is limited by insufficient penetration depth and therefore cannot access subcortical brain structures. Collectively, these modalities differ substantially in spatial resolution, temporal sensitivity, and physiological specificity, leading to inherently incomplete and partially overlapping characterizations of brain pathology. Consequently, no single modality is sufficient to comprehensively capture the multiscale and multimodal nature of complex brain disorders.

To address these limitations, a variety of multimodal neuroimaging fusion approaches have been proposed, including feature-level fusion, decision-level fusion, and deep learning-based frameworks, which have shown promising performance in disease classification, biomarker identification, and brain function analysis. However, the rapid proliferation of fMRI, EEG, fNIRS, and PET studies has led to an exponential increase in heterogeneous findings across modalities, disease categories, and experimental paradigms, resulting in increasingly fragmented neuroimaging knowledge characterized by inconsistent representations and weakly organized cross-modal relationships. Although traditional narrative reviews attempt to synthesize existing evidence, they rely heavily on manual curation and expert interpretation, making them both time-consuming and inherently subjective. More importantly, such approaches struggle to uncover latent associations across modalities and disorders, and typically produce unstructured textual summaries that are not amenable to computational querying or large-scale integration. Consequently, it remains challenging to obtain a systematic and integrative understanding of multimodal neuroimaging research, as well as to identify hidden cross-modal and cross-disease knowledge relationships.

In recent years, knowledge graphs (KGs) and large language models (LLMs) have provided complementary technical foundations for addressing these challenges. KGs offer a structured paradigm for representing scientific knowledge by transforming heterogeneous findings into relational triples (e.g., task–brain region–activation pattern), thereby enabling explicit modeling of associations across multiple dimensions of neuroimaging data [8]. In parallel, LLMs have demonstrated strong capabilities in natural language understanding and information extraction, facilitating the automatic identification of key entities such as experimental paradigms, brain regions, and activation outcomes from large-scale literature corpora [9]. For example, GraphCogent employs multi-agent collaboration to mitigate LLMs’ working-memory constraints in complex graph reasoning [10]. This work highlights the potential of LLMs for graph-related tasks, while the present study extends this perspective to the systematic construction of domain-specific knowledge graphs from heterogeneous scientific literature.

Based on these advances, and motivated by the lack of a unified framework for integrating multimodal neuroimaging evidence, we propose a knowledge discovery framework that combines LLMs with knowledge graph construction. The framework is designed to systematically organize and integrate findings across multiple imaging modalities, including fMRI, EEG, fNIRS, and PET, and to generate a structured representation of neuroimaging knowledge. By explicitly modeling relationships among experimental tasks, brain regions, disease categories, and neurophysiological signals, the proposed approach enables cross-modal knowledge alignment and supports downstream discovery of potential biomarkers and clinically relevant patterns.

First, the semantic understanding and information extraction capabilities of large language models (LLMs) were employed to identify core neuroimaging knowledge entities from the literature, including diseases, brain regions, biomarkers, imaging modalities, analytical methods, and clinical applications. In addition, semantic relationships among these entities were extracted to enable structured representation of multimodal neuroimaging knowledge in the form of a unified knowledge graph. Second, a series of network-based analyses were conducted to characterize the global organization of the constructed knowledge graph, including topological analysis, hub node identification, and community detection. These analyses were used to reveal the underlying structural and modular organization of the neuroimaging knowledge space. To further assess cross-modal interactions, a systematic cross-modal consistency analysis was performed to evaluate knowledge overlap and collaborative relationships among major imaging modalities, including fMRI, EEG, fNIRS, and PET. Finally, disease-oriented analyses were conducted using representative conditions, including Alzheimer’s disease, major depressive disorder, and chronic pain, to examine modality-specific biomarker structures and knowledge organization patterns. This analysis enabled the identification of potential biomarkers and clarified the relationships among neural mechanisms, imaging-derived features, and clinical applications within each disease context.

The main contributions of this study are as follows. First, we propose a knowledge discovery framework that integrates large language models with knowledge graphs for multimodal neuroimaging literature. This framework enables efficient extraction and organization of neuroimaging entities and their semantic relationships, providing a scalable solution for structured knowledge representation in this domain. Second, we construct a large-scale multimodal neuroimaging knowledge graph encompassing fMRI, EEG, fNIRS, and PET studies. The resulting graph provides a unified and structured representation of cross-modal neuroimaging knowledge, facilitating systematic integration of heterogeneous evidence across modalities. Third, based on the constructed knowledge graph, we perform comprehensive network-based analyses, including topology characterization, community detection, and cross-modal association analysis. These analyses reveal the underlying organizational structure of the neuroimaging field, characterize modality-specific collaboration patterns, and uncover disease-related biomarker organization. Collectively, this study establishes an integrated computational framework for organizing, analyzing, and interpreting multimodal neuroimaging knowledge, supporting future research in evidence integration, biomarker discovery, and clinical translation.

## 2. Materials and Methods

### 2.1. Data Collection and Literature Retrieval

A systematic literature retrieval strategy was adopted to identify peer-reviewed studies on multimodal neuroimaging, including functional magnetic resonance imaging (fMRI), electroencephalography (EEG), functional near-infrared spectroscopy (fNIRS), and positron emission tomography (PET). The retrieval process combined keyword-based searching, semantic expansion, citation tracking, and manual screening to maximize both the coverage and relevance of the collected literature. The literature search was conducted using four major bibliographic databases: PubMed, Web of Science, Scopus, and PsycINFO. These databases were selected because they collectively provide broad coverage of biomedical science, neuroscience, psychology, and interdisciplinary research, thereby ensuring comprehensive identification of multimodal neuroimaging studies. The use of multiple complementary databases helped reduce potential publication bias by broadening source coverage and limiting reliance on any single database.

A hierarchical search strategy was developed to systematically retrieve relevant studies (Table 1). Search terms were established through manual term identification and expert verification. Initially, core concepts and their synonymous expressions were identified based on seminal reviews, methodological guidelines, and highly cited publications in the neuroimaging field. The resulting term set was reviewed and refined to incorporate terminology variants, emerging concepts, and discipline-specific expressions used across different research communities. Within each thematic category, synonymous terms were combined using the Boolean operator “OR” to maximize retrieval sensitivity, whereas different thematic categories were linked using “AND” to enhance search specificity. The literature search covered publications from January 2016 to May 2026, providing comprehensive coverage of recent advances in multimodal neuroimaging research.

Following literature retrieval, predefined inclusion and exclusion criteria were applied to determine the final dataset. Studies were included if they (1) were peer-reviewed publications from January 2016 to May 2026, (2) employed fMRI or other relevant neuroimaging techniques, and (3) addressed topics within the scope of multimodal neuroimaging. Duplicate records, studies outside the research scope, those lacking substantive relevance to the research objectives, and those with insufficient information for reliable knowledge extraction were excluded. Title and abstract screening was followed by full-text assessment, with ambiguous cases manually reviewed to ensure consistent application of the criteria. These procedures were used to reduce potential selection bias and improve the consistency of the final dataset.

### 2.2. Knowledge Extraction Framework Based on Large Language Models

**Figure 1:**
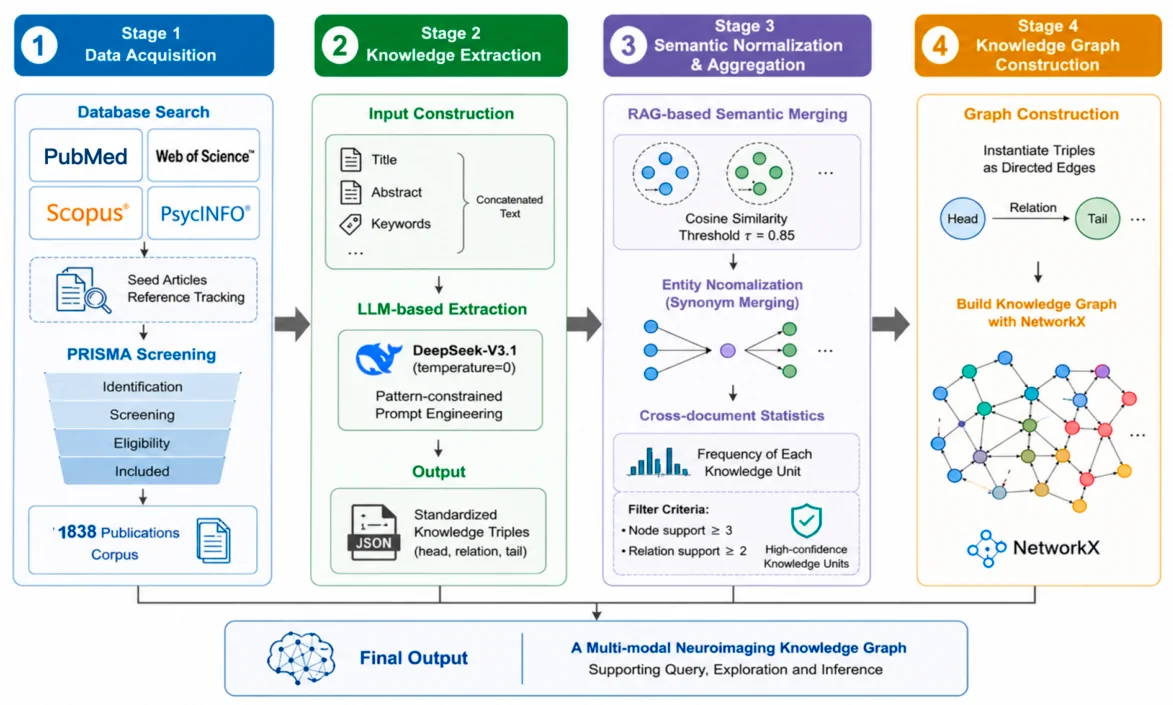
Knowledge extraction framework. Arrows indicate the sequential flow of the research process, whereas different colored circles are used to distinguish different stages of the framework.

A five-stage pipeline was developed to support the workflow from literature retrieval to knowledge graph construction and analysis (Figure 1). The pipeline consists of data acquisition, knowledge extraction, semantic merging, graph construction, and statistical analysis, ultimately generating a multimodal neuroimaging knowledge graph for structured knowledge representation and analysis.

Stage 1: Literature was retrieved from PubMed, Web of Science, Scopus, and PsycINFO using the predefined search strategy. Citation tracking was performed to supplement the initial search. After PRISMA-based screening, 1838 articles were included in the final corpus.

Stage 2: The title, abstract, and keywords of each article were concatenated as model input. A pattern-constrained prompt engineering strategy was adopted, and DeepSeek-V3.1 (temperature = 0) was used to perform entity recognition and relation extraction, generating standardized knowledge triples in JSON format.

Stage 3: A retrieval-augmented generation (RAG)-based semantic merging framework was employed to normalize synonymous entities using a cosine similarity threshold of τ= 0.85. A knowledge unit was retained if either its node support reached at least three independent literature mentions or its relation support reached at least two independent literature mentions.

Stage 4: The filtered knowledge triples were instantiated as a directed graph in NetworkX 3.2.1 to construct the multimodal neuroimaging knowledge graph.

## 3. Implementation and Results

### 3.1. Literature Search and Screening Results

Keyword-based searching alone may overlook relevant studies because of variations in terminology, inconsistent naming conventions, and the continual emergence of new concepts. To mitigate this limitation, citation tracking was employed as a complementary retrieval strategy. Specifically, the reference sections of the included studies were systematically reviewed, and citation-based screening was further performed on the most influential publications, defined as the top 10% according to citation frequency, to uncover potentially overlooked evidence. Studies that met the established eligibility criteria but were absent from the initial database search were subsequently added to the final dataset. This iterative approach served as a supplementary retrieval strategy, enhancing the coverage and robustness of the literature identification process.

The database search yielded 14,891 records (Table 2). Citation tracking identified an additional 6081 records. After merging both sources, a total of 20,972 records entered the PRISMA screening workflow. Screening and eligibility assessment were conducted in accordance with the PRISMA (Preferred Reporting Items for Systematic Reviews and Meta-Analyses) guidelines to ensure methodological transparency, reproducibility, and traceability of the literature selection process.

Given that this study aimed to characterize collaborative application patterns between functional magnetic resonance imaging (fMRI) and other neuroimaging modalities, including EEG, fNIRS, and PET, and to map the underlying knowledge structure, records were required to meet the following criteria: (1) original research articles published in peer-reviewed journals; (2) studies employing task-based experimental paradigms that combined fMRI with one or more additional neuroimaging modalities (EEG, fNIRS, or PET) to investigate brain activity under specific cognitive, emotional, or behavioral conditions; (3) explicit reporting of task-evoked neural responses, including regional activation, functional connectivity, brain network characteristics, or other neuroimaging measures, together with the corresponding brain regions, networks, or imaging features; and (4) clear descriptions of the experimental design, data processing pipeline, and statistical analysis, including significance thresholds and multiple comparison correction procedures.

Following title screening, abstract screening, and full-text assessment, 1838 records met the eligibility criteria and were retained for subsequent knowledge extraction, knowledge graph construction, and knowledge evolution analysis.

### 3.2. Implementation and Results of Knowledge Extraction

#### 3.2.1. Extraction Implementation

Document Construction and Input Preparation: The 1838 retrieved articles were first preprocessed. For each article, the following metadata fields were extracted: title, abstract, keywords, publication year, journal, authors, and DOI. These fields were concatenated to form a unified text input:

$${\mathrm{doc}}_{i}\;=\;\mathrm{concat}\;({\mathrm{title}}_{i},\;{\mathrm{abstract}}_{i},\;{\mathrm{keywords}}_{i},\;{\mathrm{year}}_{i},\;{\mathrm{journal}}_{i},\;{\mathrm{authors}}_{i},\;{\mathrm{DOI}}_{i})$$

where ${\mathrm{doc}}_{i}$ denotes the concatenated textual representation of ${\mathrm{article}}_{i}$, and ${\mathrm{title}}_{i},\;{\mathrm{abstract}}_{i},\;{\mathrm{keywords}}_{i},\;{\mathrm{year}}_{i},\;{\mathrm{journal}}_{i},\;{\mathrm{authors}}_{i}$ and ${\mathrm{DOI}}_{i}$ denote the corresponding metadata fields of ${\mathrm{article}}_{i}$. The resulting document collection D = {$d_{1}$,$d_{2}$,…,$d_{n}$} retained the core semantic information relevant to brain functional neuroimaging research. This included regional activation patterns, experimental task paradigms, subject population characteristics, disease states, and descriptions of neural mechanisms. This collection served as the primary input for subsequent knowledge extraction.

Prompt Engineering and Schema Definition: To improve the consistency and controllability of knowledge extraction, a schema-constrained prompt engineering strategy was adopted. The model was restricted to performing entity recognition and relation extraction within a predefined knowledge schema, thereby transforming open-ended knowledge discovery into a structured information extraction task. The system prompt comprised four components: role definition, domain knowledge injection, schema constraints, and output format constraints. Specifically, the model was instructed to act as an expert in neuroimaging knowledge graph construction. Domain knowledge describing multimodal neuroimaging techniques and their clinical applications was provided as contextual guidance. Schema constraints predefined the allowable entity categories and relation types, while output format constraints required all extraction results to be returned in a standardized JSON format. The core components of the prompt design are summarized in Table 3.

Entity and Relation Extraction: Following document construction and prompt definition, the DeepSeek-V3.1 large language model was employed to perform structured entity and relation extraction for each article. The temperature parameter was fixed at 0 to ensure deterministic and reproducible outputs. The extraction process is formulated as follows:

(1) $$F_{LLM}({doc}_{i})\to (N_{i},R_{i})$$

where $doc_{i}$ denotes the text of the article, $N_{i}$ denotes the extracted entity set, and $R_{i}$ denotes the extracted relation set. A knowledge triple space is further defined, where ε denotes the full entity set aggregated across all articles, R denotes the full relation set aggregated across all articles, and h, r and t denote the head entity, relation, and tail entity of a triple, respectively:

(2) $${\;T}_{i}=\{(h,r,t)|h\in \varepsilon ,\;r\in R,\;t\in \varepsilon \}$$

where $T_{i}$ denotes the set of knowledge triples extracted from $article_{i}$. Based on the characteristics of multimodal neuroimaging research, a unified schema of entity and relation types was defined (Table 4 and Table 5). As summarized in Table 4, six entity categories were included. Neuroimaging Modality represents imaging techniques such as fMRI, EEG, fNIRS, and PET. The brain region includes neuroanatomical structures, such as the hippocampus, prefrontal cortex, and amygdala. Clinical Condition covers neurological and psychiatric disorders, including Alzheimer’s disease, depressive disorder, and Parkinson’s disease. Diagnostic Marker represents multimodal biomarkers used for disease detection and diagnosis. Treatment targets include therapeutic intervention targets, such as transcranial magnetic stimulation (TMS) and deep brain stimulation (DBS) targets. Fusion Method describes multimodal integration approaches, including EEG-informed fMRI and fMRI-guided fNIRS. Together, these entity categories provide a structured semantic framework spanning neuroimaging techniques, brain structures, clinical conditions, biomarkers, therapeutic targets, and multimodal fusion methods.

To ensure consistency in relation extraction, a unified definition was established for each relation type (Table 5). DIAGNOSTICALLY SYNERGIZES refers to the improvement in disease diagnosis or classification achieved through the combined use of multiple neuroimaging modalities. SPATIALLY COMPLEMENTS and TEMPORALLY INFORMS describe the complementary contributions of different modalities to spatial and temporal information, respectively. FUSES WITH represents the integration of multiple neuroimaging modalities at the levels of data acquisition, feature extraction, or data analysis. TREATMENT GUIDES indicates that neuroimaging evidence supports treatment target localization or therapeutic efficacy evaluation. CONVERGES WITH denotes that different modalities provide consistent or mutually reinforcing evidence regarding the same disease, brain region, or neural mechanism. CONTRADICTS indicates that different modalities or independent studies report inconsistent or conflicting findings concerning the same research topic. All extracted relations were required to be supported by explicit evidence from the source literature and to conform to the predefined relation schema.

RAG-Based Entity Normalization and Semantic Consolidation: Terminological variation across studies means that the same brain region, disease, or imaging technology is often referred to by multiple name variants. To improve cross-literature knowledge integration, a Retrieval-Augmented Generation (RAG) mechanism combined with a semantic consolidation strategy was introduced. During the processing of each article, high-frequency entities and representative relations already extracted from previously processed articles were dynamically retrieved and injected into the prompt as contextual information. This guided the model to maintain terminological consistency throughout the subsequent extraction process.

For each newly extracted entity $e_{new}$, the system computed its cosine similarity against every entity $E=\{e_{1},e_{2},\ldots ,e_{n}\}$ in the existing entity repository:

(3) $$sim(e_{new},e_{i})=\frac{v_{new}\cdot v_{i}}{\left\|v_{new}\|\cdot \|v_{i}\right\|}$$

where the two vectors $v_{new}$ and $v_{i}$ represent the embedding representations of the two entities encoded by the embedding model. The consolidation decision rule is defined as follows:

(4) $$e_{new}\to \{\begin{array}{c} e^{*}=\arg\underset{e_{i}\in E}{\max}\;sim(e_{new},e_{i})\;\mathrm{if}\;\underset{e_{i}\in E}{\max}\;\mathrm{sim}(e_{new},e_{i})\geq \tau \\ e_{new}(\mathrm{New}\;\mathrm{Node})\;\;\;\;\;\;\;\;\;\;\;\;\;\;\;\;\;\;\;\;\;\;\;\;\;\;\;\;\;\;\;\;\;\;\;\;\;\;\;\mathrm{otherwise} \end{array}$$

Following the findings of Wang et al. (2025) [10], which indicated that semantic similarity thresholds around 0.8 provided a favorable balance between entity consolidation and semantic distinction, we adopted a slightly more conservative threshold of 0.85 to ensure more rigorous entity consolidation and minimize the risk of merging semantically distinct entities. Here, τ=0.85 denotes the predefined similarity threshold, and $e^{*}$ denotes the existing standard entity with the highest semantic similarity to the new entity e. When the similarity meets or exceeds the threshold, the new entity $e_{new}$ is consolidated into $e^{*}$ and no new node is added; otherwise, it is added to the entity repository as a new node. This mechanism enables dynamic standardization of knowledge units across literature without relying on manually maintained dictionaries. It reduces node fragmentation caused by synonymous expressions and improves the overall connectivity and knowledge aggregation capacity of the graph.

#### 3.2.2. Reliability Assurance

To improve the standardization and reproducibility of LLM-based knowledge extraction, we developed a structured prompting framework that constrained the extraction process in terms of role specification, language conventions, entity and relation definitions and output format (Table 6). This framework was designed to reduce semantic ambiguity and classification errors arising from unconstrained generation.

Under the structured prompting framework, the model performed knowledge extraction sequentially through language checking, entity extraction, relation extraction, quantity filtering, and structured output. Entities or relations not covered by the predefined schema but possessing clear semantic relevance were permitted through controlled schema extension and were classified according to the established naming and categorization rules. This approach maintained extraction consistency while minimizing the loss of information from the source literature.

To address potential hallucinations, entity misidentification, and relation classification errors, a multilayer quality-control strategy combining structured prompting, automated post-processing, and manual validation was implemented. First, entity types, relation types, directions, evidence levels, and output formats were predefined to reduce semantic and structural deviations from unconstrained generation. Second, model outputs underwent semantic normalization and frequency-based filtering: semantically equivalent entities were merged using similarity measures and synonym-mapping rules, while knowledge units with insufficient independent literature support were removed based on their document-level support frequency. These procedures reduced redundancy and the influence of potentially spurious knowledge on graph structure. Finally, to assess the accuracy of the extracted knowledge, 50 articles were randomly sampled from the 1838 included studies for manual validation. Two independent coders with expertise in neuroimaging and related brain science independently reviewed the extracted nodes, relations, and their semantic type assignments. Each item was classified as accurate or inaccurate according to whether it had explicit support in the source text and whether its semantic meaning and type assignment were consistent with the original content (Table 7). Of the 144 nodes evaluated, 127 were supported by the source literature, yielding a node extraction precision of 88.2%. Of the 78 relations evaluated, 67 were supported, corresponding to a relation extraction precision of 85.6%. The two coders achieved a Cohen’s κ = 0.76, indicating high inter-coder agreement. These results demonstrate the reliability of the knowledge extraction pipeline and its systematic quality-control procedures.

#### 3.2.3. Extraction Results

DeepSeek-V3.1 was invoked once for each of the 1838 articles to perform structured knowledge extraction, with the temperature parameter fixed at 0 to ensure deterministic outputs. The JSON parsing success rate reached 99.18%, and extraction results from 1823 articles were successfully parsed and retained for subsequent analysis. In total, 21,951 raw nodes and 17,259 raw relations were extracted. Given that these raw extraction results may contain sporadically occurring knowledge elements and unstable associations, a frequency-based pruning procedure was further conducted to determine appropriate support thresholds.

Following synonym normalization, the occurrence frequencies of candidate nodes and relations across the 1838 articles were calculated (Table 8), and support thresholds were determined by comparing the number of knowledge elements retained at different frequency cutoffs. For nodes, 80.91% of candidate nodes occurred only once or twice, likely representing incidental mentions, context-specific findings, or concepts with limited literature support. Retaining these low-frequency nodes could introduce substantial noise into the knowledge graph. A minimum node support threshold of ≥3 retained 4190 nodes (19.08%), whereas increasing the threshold to ≥4 reduced the number of retained nodes to 1999 (9.11%), resulting in a substantial loss of knowledge coverage. Accordingly, a node support threshold of ≥3 was adopted to exclude weakly supported concepts while preserving nodes with sufficient evidence across the literature.

For relations, 59.40% occurred only once, indicating that single-occurrence associations were prevalent among the extracted candidate relations. Applying a minimum relation support threshold of ≥2 retained 7007 relations (40.60%), thereby removing potentially incidental associations while maintaining a broader range of reported relationships. Increasing the threshold to ≥3 reduced the number of retained relations to 3576 (20.72%), resulting in a considerable reduction in relation coverage. Based on these comparisons, a dual filtering criterion of node support ≥3 and relation support ≥2 was adopted. This criterion provided a balance between excluding weakly supported knowledge elements and retaining potentially informative associations with relatively limited but repeated literature support.

After threshold-based pruning, using a minimum node support of 3 and a minimum relation support of 2, the graph was reduced to 4190 nodes (19.08% retention) and 7007 relations (40.60% retention), forming the final high-confidence knowledge graph. A comparison of the graph scale before and after pruning is presented in Table 9.

Regarding the distribution of relation types, the extracted knowledge graph contained seven predefined semantic relation categories (Table 10). DIAGNOSTICALLY SYNERGIZES was the most prevalent relation, with 2881 instances (41.1%), indicating that multimodal integration for disease diagnosis represents the dominant research focus in the current literature. FUSES WITH (1464 instances, 20.9%) and SPATIALLY COMPLEMENTS (968 instances, 13.8%) ranked second and third, reflecting the importance of methodological integration and the complementary spatial characteristics of different neuroimaging modalities. CONVERGES WITH accounted for 978 instances (14.0%), capturing consistent findings across modalities and highlighting the role of cross-modal evidence integration in validating neuroimaging results. A total of 344 CONTRADICTS relations (4.9%) were identified, primarily involving PET-related and multimodal-related nodes. These contradictions likely reflect differences in imaging tracers, data acquisition protocols, or analytical pipelines across studies, suggesting that evidence heterogeneity remains a challenge in multimodal neuroimaging research. By contrast, TREATMENT GUIDES (226 instances, 3.2%) and TEMPORALLY INFORMS (144 instances, 2.1%) were the least frequent relation types, corresponding to treatment target identification and temporal complementarity. Their relatively low frequencies suggest that these research directions remain less explored than diagnostic applications.

Regarding the entity category distribution, Diagnostic Marker constituted the largest entity category, with 1412 nodes accounting for 33.70% of all entities. Brain Region (1008 nodes, 24.10%) and Fusion Method (945 nodes, 22.60%) ranked second and third, respectively. Together, these three categories accounted for 80.40% of all entities, indicating that the knowledge graph is primarily organized around diagnostic biomarkers, neuroanatomical structures, and multimodal fusion methodologies. Modality comprised 644 nodes (15.40%), whereas Treatment Target included 144 nodes (3.40%). The relatively small proportion of Treatment Target nodes suggests that knowledge related to treatment-oriented applications remains less extensively represented in the current literature.

### 3.3. Implementation and Topological Analysis of Knowledge Graph

#### 3.3.1. Knowledge Graph Instantiation and Visualization

In this step, a directed graph $G^{*}=(V^{*},E^{*})$ was instantiated using the DiGraph() class in NetworkX 3.2.1, where $V^{*}$ denotes the node set and $E^{*}$ denotes the edge set. Each node stored four attributes: entity type, neuroimaging modality, entity category, and literature support frequency. Each edge stored the corresponding relation type, fusion type, cross-modal identifier, and literature support frequency. The graph construction process is formulated as follows:

(5) $$G.add\_edge(h,\;t,\;label=r,\;freq=w(h,r,t),\;task\_category=T_{h,r,t},\;clinical\_contexts=C_{h,r,t})\;$$

where h and t denote the head and tail entities of a triple, r denotes the relation type linking h and t, w(h,r,t) denotes the number of articles supporting the triple (h,r,t), $T_{h,r,t}$ denotes the task or disease category associated with the triple, and $C_{h,r,t}$ denotes the context(s) in which the triple was reported. This operation integrated the extracted entities and relations into a unified graph structure while preserving their semantic associations. Literature support frequencies were explicitly encoded as edge attributes, enabling subsequent evidence-weighted network analysis and graph visualization. To improve the interpretability of the resulting knowledge graph, evidence-weighted visualization was applied by jointly encoding graph topology and edge support frequencies. The graph layout was optimized using the Fruchterman–Reingold force-directed algorithm:

(6) $$E=\sum_{(i,j)\in E}\frac{\|x_{i}-x_{j}{\|}^{2}}{k}-\sum_{i<j}\frac{k}{\left\|x_{i}-x_{j}\right\|}$$

where $x_{i}$ and $x_{j}$ denote the coordinates of nodes i and j, respectively, and k is the ideal edge length parameter. The first term represents the attractive force between connected nodes, whereas the second term represents the repulsive force between all node pairs. By iteratively balancing these attractive and repulsive forces, the algorithm generated a layout that preserved the graph topology while improving the spatial separation of nodes. Static high-resolution network visualizations were generated using NetworkX 3.2.1, and interactive knowledge graph visualizations were developed using PyVis 0.3.2 to facilitate dynamic exploration of the graph structure and evidence path tracing.

#### 3.3.2. Topological Feature Analysis

In this study, knowledge maturity is characterized using structural properties of the knowledge network within each neuroimaging research domain. Network density and modular organization serve as the primary indicators, complemented by degree distribution, clustering coefficient, degree centrality, and betweenness centrality. These measures collectively characterize network connectivity, community structure, connectivity heterogeneity, local cohesion, and the structural importance of key knowledge units, respectively.

As shown in Table 11, the constructed neuroimaging knowledge graph comprised 4190 nodes and 7007 relations, with an overall graph density of 3.99 × 10^−4^, indicating a sparse network structure. The graph contained 783 connected components, including 629 single-node isolated components and 108 two-node components. These results indicate that the knowledge graph contained a number of small or isolated components in addition to its larger connected structures.

The connectivity structure was dominated by the weakly connected components. The largest WCC contained 3209 nodes, corresponding to a WCC coverage rate of 76.60%, and included 6736 internal edges, accounting for 96.10% of all graph edges. Its density was 6.54 × 10^−4^, compared with an overall graph density of 3.99 × 10^−4^, while the average degree within the WCC was 4.2. These results show that most nodes and relations were concentrated within weakly connected structures, particularly the largest WCC.

To identify the key knowledge units within the knowledge graph, node degree and betweenness centrality were calculated as topological measures, and the highest-ranked hub nodes were subsequently identified.

Degree distribution analysis: The in-degree $k_{in}(v)$ and out-degree $k_{out}(v)$ of each node were computed to identify high-connectivity hub nodes:

(7) $$k(v)=k_{in}(v)+k_{out}(v)$$

Betweenness centrality: This metric measures the bridging role of a node along knowledge propagation paths:

(8) $$B(v)=\sum_{s\neq v\neq t}\frac{\sigma_{st}(v)}{\sigma_{st}}$$

where $\sigma_{st}$ denotes the total number of shortest paths from node s to node t, and $\sigma_{st}(v)$ denotes the number of those shortest paths that pass through node v.

Clustering coefficient: This metric measures the connection density among a node’s neighbors, reflecting the internal cohesion of functional knowledge clusters:

(9) $$Clusc(v)=\frac{\left|\{e_{jk}:v_{j},v_{k}\in N(v),e_{jk}\in E\}\right|}{k(v)(k(v)-1)}$$

where N(v) denotes the set of neighboring nodes of v, $e_{jk}$ denotes an edge connecting neighbors $v_{j}$ and $v_{k}$, E denotes the edge set of the graph, and k(v) denotes the degree of node v as defined in Equation (7).

Eigenvector centrality: This metric measures the structural influence of a node by considering not only its direct connections but also the centrality of its neighboring nodes, thereby assigning greater importance to connections with highly central nodes:

(10) $$\;Ec\;(v)=\frac{1}{\lambda }\sum_{u\in N(v)}A_{vu}x_{u}$$

where N(v) denotes the set of neighboring nodes of v, $A_{vu}$ denotes the adjacency matrix entry between nodes v and u, $x_{u}$ denotes the eigenvector centrality of node u, and λ denotes the largest eigenvalue of the adjacency matrix.

Regarding the composition of hub nodes (Table 12), the highest-ranking nodes were predominantly associated with Alzheimer’s disease, neuroimaging modalities, and disease diagnosis. Alzheimer’s disease exhibited the highest k(v) = 767, B(v) = 0.2267, Ec(v) = 0.1461, and Clusc(v) = 0.1057, indicating its dominant role within the knowledge network. Disease-related nodes, including mild cognitive impairment (MCI) and major depressive disorder, were identified as important hubs, while neuroimaging modalities such as fMRI, EEG, amyloid-β PET, FDG-PET, and resting-state fMRI also ranked among the most central nodes.

Among the identified neuroimaging modalities, fMRI exhibited a prominent network position, with a k(v) of 366, a B(v) of 0.1037, an Ec(v) of 0.0867, and a Clusc(v) of 0.1029. This was followed by amyloid-β PET (k(v) = 320, B(v) = 0.0649, Ec(v) = 0.0623, Clusc(v) = 0.0813), FDG-PET (Degree = 256, B(v) = 0.0515, Ec(v) = 0.0231, Clusc(v) = 0.0992), EEG (k(v) = 236, B(v) = 0.0772, Ec(v) = 0.0168, Clusc(v) = 0.0874), and resting-state fMRI (k(v) = 151, B(v) = 0.0264, Ec(v) = 0.1122, Clusc(v) = 0.1053). The additional centrality measures provide a more comprehensive characterization of node influence and network proximity while remaining consistent with the original interpretation based on degree and betweenness centrality.

Collectively, these findings indicate that the constructed knowledge graph is primarily organized around Alzheimer’s disease and multimodal neuroimaging techniques, reflecting the central roles of MRI-, PET-, and EEG-based evidence within the extracted knowledge network. Major depressive disorder (k(v) = 198, B(v) = 0.0488, Ec(v) = 0.0742, Clusc(v) = 0.0980) was the only non-neurodegenerative disease represented among the major hub nodes. However, its centrality measures remained substantially lower than those of Alzheimer’s disease, suggesting that psychiatric disorders are comparatively less represented than neurodegenerative diseases within the current knowledge graph.

#### 3.3.3. Analysis of the Largest Connected Component

**Figure 2:**
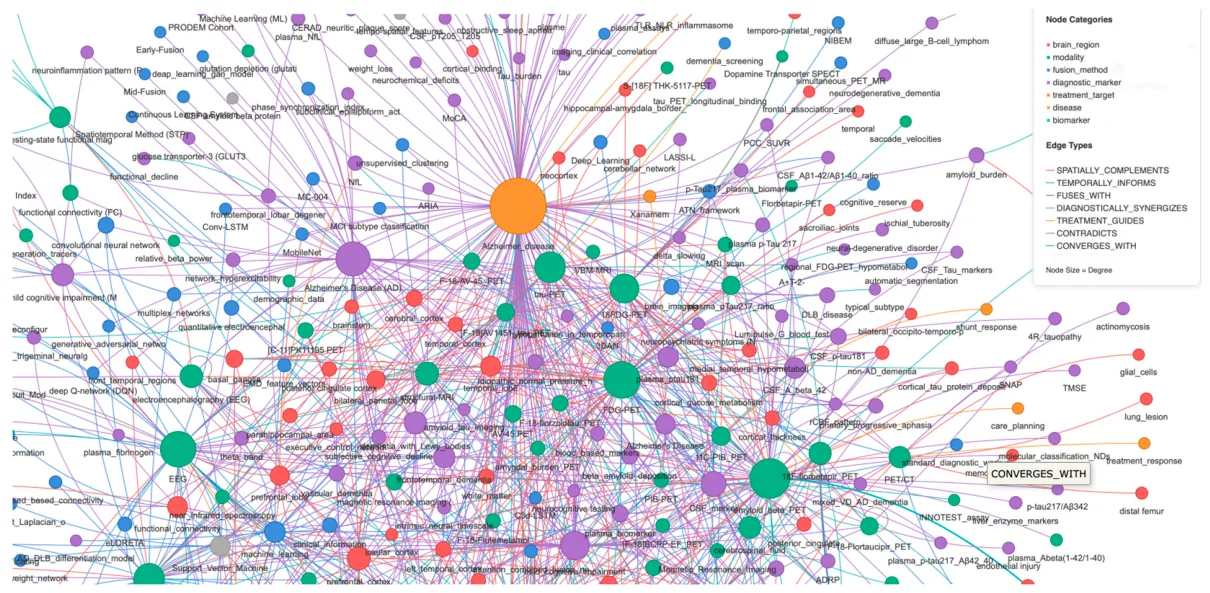
Largest connected component.

Connectivity analysis identified a dominant weakly connected component (WCC) within the knowledge graph. As shown in Figure 2, the largest WCC comprised 3209 nodes, representing a substantial proportion of the entire graph. Its network density was 0.000654, higher than the overall graph density of 0.000399, and its mean k(v) was 4.20 (Table 11). These characteristics indicate that the largest WCC constituted the principal connected subnetwork within the knowledge graph.

The concentration of relationships within connected components was further reflected by the WCC edge proportion of 96.10%, indicating that the vast majority of relations were contained within weakly connected structures. By comparison, the remaining components were considerably smaller and contributed fewer connections to the overall network. Taken together, these findings demonstrate a concentrated connectivity structure, with the largest WCC accounting for the primary connected portion of the knowledge graph.

#### 3.3.4. Modality Subgraph Analysis

To characterize how different neuroimaging modalities organize knowledge, we computed topological metrics-node count, network density, and clustering coefficient—for each modality-specific subgraph (Table 13).

Among the modality-specific subgraphs, the multimodal subgraph was the largest, comprising 2126 nodes, yet it exhibited the lowest network density (0.0003) and a relatively low clustering coefficient (0.1824). These characteristics suggest that the multimodal subgraph functions primarily as an integrative scaffold that bridges knowledge across different imaging modalities, rather than forming a densely interconnected structure in its own right. By contrast, the fNIRS subgraph, despite containing only 76 nodes, exhibited the highest network density (0.0105) and clustering coefficient (0.4135) among all modalities, reflecting a tightly interconnected internal structure with strong local cohesion among its constituent concepts. This pattern likely reflects the relatively focused research topics, more homogeneous study populations, and greater methodological consistency characteristic of fNIRS research. The fMRI (clustering coefficient = 0.2947) and PET (clustering coefficient = 0.2209) subgraphs occupied an intermediate position, indicating moderate local connectivity within their respective knowledge structures. Notably, although the EEG subgraph contained 454 nodes, comparable in scale to the fMRI and PET subgraphs, its clustering coefficient was only 0.0281, the lowest among all major modalities.

### 3.4. Community Detection: Implementation and Results

#### 3.4.1. Brain Region Hub Identification

**Figure 3:**
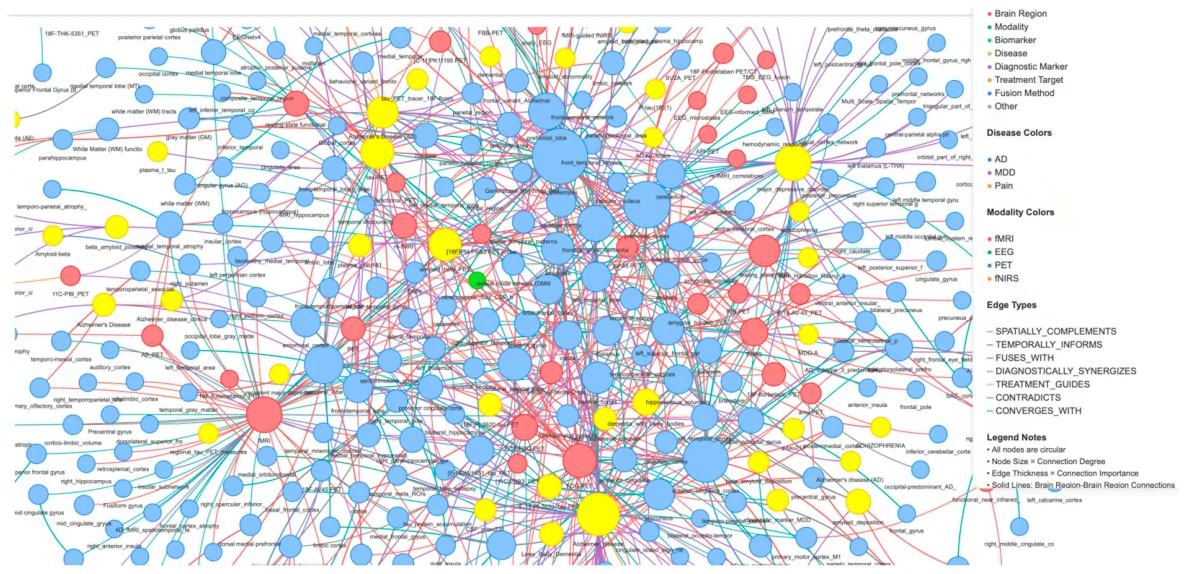
Core brain region hubs.

To identify key anatomical hubs within the multimodal neuroimaging knowledge network, brain region nodes were analyzed based on node degree and cross-modal connectivity (Table 14). As shown in Figure 3, the hippocampus (k(v) = 121), prefrontal cortex (k(v) = 63), and anterior cingulate cortex (k(v) = 56) exhibited the highest connectivity, forming the principal anatomical hubs of the knowledge graph.

Among these regions, the hippocampus represented the most highly connected node, with 68 cross-modal links spanning six evidence modalities: fMRI, PET, EEG, MRI, fNIRS, and blood biomarkers. Its dominant relationship types were SPATIALLY COMPLEMENTS (46 instances) and CONVERGES WITH (33 instances), indicating that multimodal evidence primarily characterizes hippocampal function through spatial complementarity and cross-modal validation. As a critical brain region involved in learning, memory, cognitive processing, and neurodegenerative disorders, the prominent position of the hippocampus within the network is consistent with its central role in studies of Alzheimer’s disease, mild cognitive impairment (MCI), and related neurological disorders.

The prefrontal cortex and anterior cingulate cortex also emerged as major anatomical hubs within the knowledge network. The prefrontal cortex integrated evidence from five modalities, including fMRI, EEG, PET, fNIRS, and TMS, with SPATIALLY COMPLEMENTS and CONVERGES WITH remaining the dominant relationship types. This distribution suggests that multimodal neuroimaging provides complementary evidence for prefrontal cortex function by integrating functional, electrophysiological, and metabolic information. Given its established role in cognitive control, executive function, and emotional regulation, the extensive cross-modal connectivity of the prefrontal cortex highlights its importance in multimodal neuroimaging research. In contrast, although the anterior cingulate cortex exhibited fewer cross-modal connections than the hippocampus, it showed slightly more CONVERGES WITH relationships than SPATIALLY COMPLEMENTS relationships. This pattern indicates a relatively high level of agreement among different neuroimaging modalities regarding this region.

#### 3.4.2. Louvain Community Detection

To reveal the underlying organizational structure of the multimodal neuroimaging knowledge network, the Louvain community detection algorithm [10] was applied to the largest weakly connected component (WCC). The algorithm detects community structures by optimizing network modularity (Q), generating clusters characterized by strong internal associations and relatively weak links across different communities. Owing to its computational efficiency and robustness, it is widely used for community detection in large-scale sparse networks. Community detection was performed on an undirected weighted graph, where edge weights were defined by the co-occurrence frequency of node pairs across all included articles. A fixed random seed of 42 was used to ensure reproducibility.

Community themes were interpreted based on dominant disease-related nodes, neuroimaging modalities, and internal relation patterns, with thematic labels derived from highly connected entities and predominant semantic relationships within each community (Table 15). Candidate thematic labels were independently reviewed by the research team, including the two corresponding authors with expertise in multimodal neuroimaging, based on the composition of hub nodes and dominant relation types. Final labels were determined through consensus discussion.

**Figure 4:**
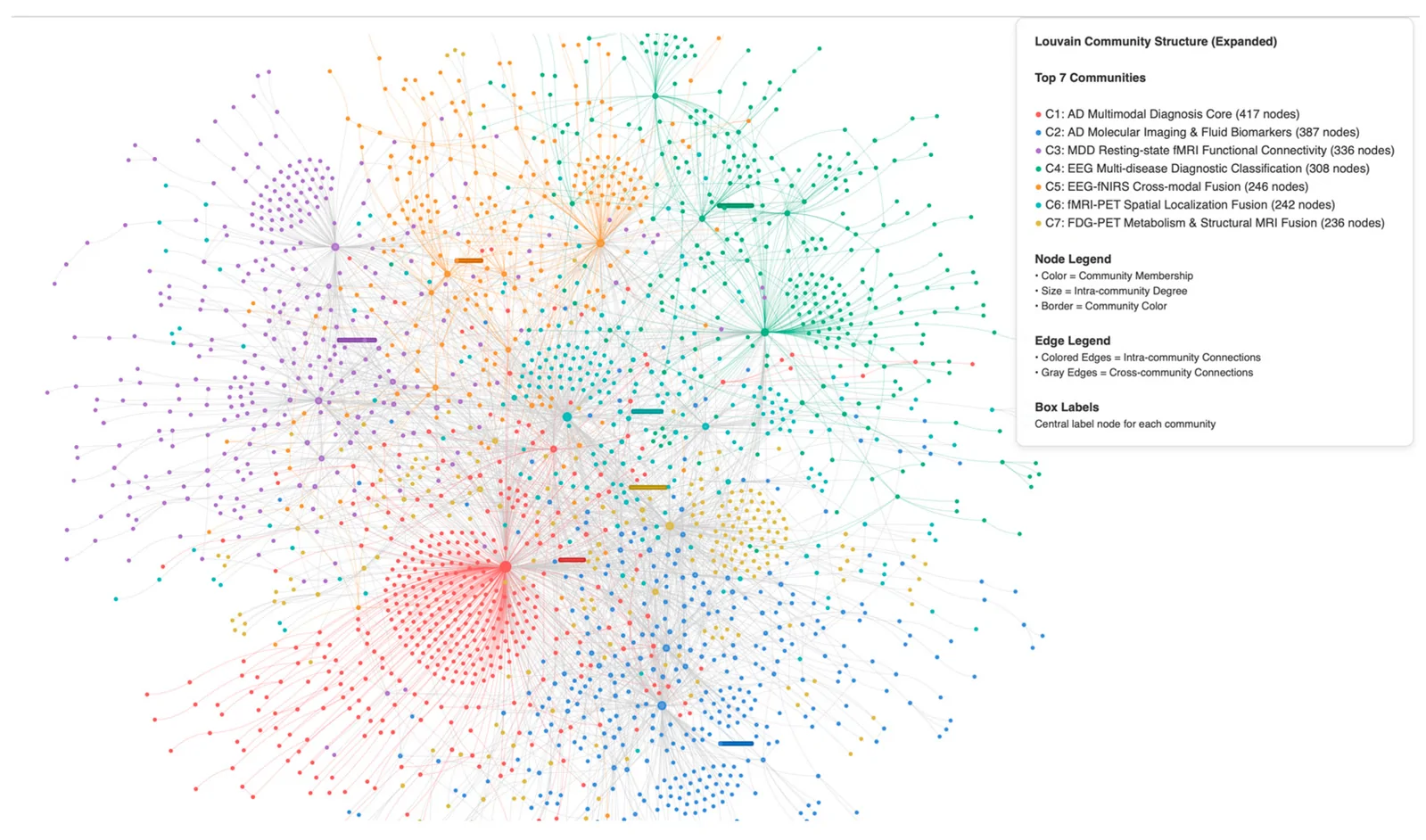
Major functional knowledge clusters.

Community detection identified 25 knowledge communities within the largest WCC, with a modularity of 0.5525, indicating a well-defined modular structure (Figure 4). The seven largest communities contained 238–422 nodes and collectively accounted for 62.6% of all nodes, representing the dominant organizational units of the knowledge graph. These communities primarily encompassed three major research themes: neurodegenerative diseases, psychiatric disorders, and multimodal neuroimaging methodologies. The remaining communities were comparatively small and mainly represented specialized topics, including specific diseases, brain regions, and methodological developments. The thematic characteristics of the seven largest communities are described in the following sections.

##### Community 1: Core Knowledge Cluster for Multimodal Diagnosis of Alzheimer’s Disease

Community 1 comprised 422 nodes and 523 internal connections, making it the largest community within the knowledge graph. Alzheimer’s disease served as the central hub, while mild cognitive impairment (MCI), amyloid-β42, and Lewy body dementia functioned as secondary connectivity hubs. The community was organized around Alzheimer’s disease and related cognitive disorders, encompassing 47 disease-associated entities that collectively represented disease diagnosis, pathological characterization, and disease progression. Analysis of the relationship composition showed that DIAGNOSTICALLY SYNERGIZES was the predominant relationship type, accounting for 67.1% of all internal relationships. Furthermore, diagnostically synergistic fusion edges represented 82.4% of all internal connections, indicating that multimodal diagnostic integration constitutes the principal organizational mechanism of this community.

The knowledge organization within this community illustrates a typical multimodal diagnostic workflow for Alzheimer’s disease. In patients with MCI, structural MRI provides evidence of neurodegenerative changes, including brain volume reduction and hippocampal atrophy. Amyloid-β PET complements these findings by detecting cerebral amyloid deposition and providing pathological evidence, whereas EEG characterizes alterations in neural activity and electrophysiological rhythms that reflect functional impairment. Together, the integration of structural, pathological, and functional evidence enables more comprehensive disease characterization than any single imaging modality alone. Collectively, these findings demonstrate that Community 1 represents the primary knowledge module for multimodal Alzheimer’s disease diagnosis, highlighting the central role of multimodal evidence integration in current diagnostic research.

##### Community 2: Molecular Imaging and Liquid Biomarker Knowledge Cluster for Alzheimer’s Disease

Community 2 comprised 391 nodes and 695 internal connections, making it one of the most densely connected communities in the knowledge graph. Unlike Community 1, which focused on multimodal diagnosis, this community was organized around Alzheimer’s disease pathology and biomarker research. Amyloid-β PET and tau-PET served as the principal hubs, while CSF Aβ42, plasma p-tau181, and p-tau217 formed important secondary nodes. Multimodal research entities accounted for 54.0% of the community, and DIAGNOSTICALLY SYNERGIZES, CONVERGES WITH, and SPATIALLY COMPLEMENTS were the dominant relationship types, reflecting extensive interactions between molecular imaging and fluid biomarkers.

From a knowledge organization perspective, this community closely corresponds to the AT(N) biological framework, which has become a widely adopted model for Alzheimer’s disease research. Within this framework, amyloid-β PET characterizes cerebral amyloid deposition, whereas tau-PET evaluates the distribution of tau pathology. Fluid biomarkers, including CSF Aβ42, p-tau181, and p-tau217, provide complementary pathological evidence from cerebrospinal fluid or peripheral blood. The co-occurrence of these entities within the same community reflects the close integration of molecular imaging and fluid biomarkers in contemporary Alzheimer’s disease research. This knowledge organization also mirrors the emerging stratified diagnostic strategy of “screening followed by confirmation.” In this framework, blood biomarkers, particularly p-tau217 and p-tau181, provide a cost-effective approach for large-scale population screening, whereas amyloid-β PET and tau-PET offer highly specific in vivo evidence of pathological protein deposition. Their complementary use supports early disease identification while improving diagnostic efficiency and reducing screening costs.

##### Community 3: Resting-State Functional Connectivity Knowledge Cluster for Major Depressive Disorder

Community 3 comprised 341 nodes and 520 internal connections, representing the primary knowledge cluster for psychiatric disorder research. Major depressive disorder and resting-state fMRI served as the principal hubs, while the default mode network (DMN), salience network (SN), prefrontal cortex, and cerebellum formed important secondary nodes. The community was characterized by a strong emphasis on brain functional networks associated with depressive disorders. Relationship analysis showed that DIAGNOSTICALLY SYNERGIZES and SPATIALLY COMPLEMENTS were the dominant relationship types. Notably, FUSES WITH relationships accounted for 26.7% of all internal connections, a substantially higher proportion than that observed in the Alzheimer’s disease-related communities, indicating a greater emphasis on multimodal data fusion in psychiatric neuroimaging research.

The clinical diagnosis of depressive disorders remains largely dependent on symptom-based assessment and standardized rating scales, with few objective biomarkers available to support early diagnosis or subtype differentiation. The multimodal fusion pattern identified in this study highlights a potential framework for addressing these limitations. Functional MRI may provide objective markers of large-scale network dysfunction, particularly within the DMN and SN, whereas EEG offers complementary temporal information on the neural dynamics underlying emotional regulation and cognitive control. Integrating these modalities across longitudinal assessments may improve treatment monitoring, facilitate prediction of relapse risk, and ultimately contribute to precision diagnosis and individualized management of depressive disorders.

##### Community 4: EEG-Based Cross-Disease Intelligent Diagnosis Knowledge Cluster

Community 4 comprised 310 nodes and 410 internal connections, representing a knowledge cluster centered on electroencephalography (EEG). Alzheimer’s disease (AD), electroencephalography (EEG), and mild cognitive impairment (MCI) served as the principal hubs, with additional links to major depressive disorder, reflecting a cross-disease knowledge organization pattern. Relationship analysis indicated that DIAGNOSTICALLY SYNERGIZES and FUSES WITH were the dominant association types, with a relatively high proportion of spatiotemporal fusion relationships. Overall, this community highlighted the growing role of EEG as a multimodal neuroimaging modality across both neurodegenerative and psychiatric disorders.

From a diagnostic perspective, although different diseases exhibit distinct pathological mechanisms, their relevant abnormal signatures are often embedded within high-dimensional, complex, and dynamically evolving EEG signals, which traditional analytical approaches struggle to fully exploit. In this context, the strong presence of artificial intelligence–related nodes, particularly convolutional neural networks (CNNs), within the community reflects a clear paradigm shift from conventional handcrafted feature engineering toward data-driven, intelligent analytical frameworks. Machine learning and deep learning methods enable the extraction of disease-associated time–frequency features, connectivity patterns, and spatial representations from large-scale EEG data, contributing to more reliable and generalizable diagnostic models. Overall, the structural patterns observed in Community 4 suggest that the deep integration of EEG with artificial intelligence is emerging as a key technological pathway for early disease screening and computer-aided diagnosis, offering the potential to deliver more cost-effective, accessible, and scalable diagnostic tools for primary healthcare and large-scale population screening applications.

##### Community 5: EEG–fNIRS Cross-Modal Fusion Knowledge Cluster

Community 5 comprised 249 nodes and 337 internal connections, representing a knowledge cluster centered on EEG–fNIRS multimodal fusion. EEG and fNIRS served as the two principal modality hubs, with intra-cluster connectivity degrees of 113 and 56, respectively. The prefrontal cortex and functional connectivity constituted the dominant brain region and functional feature nodes, while machine learning and support vector machine (SVM) represented the core methodological nodes. Relationship analysis revealed that FUSES WITH accounted for 35.9% of internal connections, substantially higher than in other major communities, with spatiotemporal fusion and temporal information fusion relations contributing 19.3% and 12.8%.

From a clinical diagnostic perspective, this community points toward a diagnostic framework oriented to practical screening scenarios. In depressive disorder research, EEG can capture abnormal neural oscillatory activity, while fNIRS can detect insufficient prefrontal cortex activation. In Alzheimer’s disease research, EEG reflects reduced neural network synchronization, while fNIRS reveals attenuated cerebral blood flow responses during cognitive tasks. Although these two signals originate from distinct physiological levels, both capture disease-related brain functional abnormalities, and thus offer strong complementary diagnostic value. In clinical settings where large-scale imaging modalities such as PET and MRI are difficult to access, the combination of EEG and fNIRS could enable the establishment of a low-cost, portable joint screening approach, supporting early identification and dynamic monitoring of neuropsychiatric disorders.

##### Community 6: fMRI–PET Spatial Complementarity Fusion Knowledge Cluster

Community 6 comprised 244 nodes and 346 internal connections, representing a knowledge cluster centered on the integration of fMRI and PET neuroimaging modalities. fMRI and PET served as the principal hubs, with cross-modal connections accounting for 72.3% of all internal links. Brain region nodes constituted the largest node category at 29.5%, exceeding diagnostic marker nodes at 23.8%, reflecting a strong emphasis on functional brain localization and regional activity mapping. Relationship analysis revealed that FUSES WITH, SPATIALLY COMPLEMENTS, and CONVERGES WITH were the dominant association types, accounting for 31.5%, 21.4%, and 14.2% of internal relations. Spatial complementarity relations collectively accounted for 41.3% of all internal connections, indicating that cross-modal spatial integration between metabolic and functional neuroimaging information constituted the primary knowledge organization principle within this community.

In clinical practice, the fMRI–PET knowledge cluster can support clinicians in characterizing the biological processes underlying disease onset and progression. When PET demonstrates increased amyloid deposition alongside fMRI evidence of reduced functional connectivity in related brain networks, clinicians can not only determine disease presence but also estimate the likely pathological stage and identify which functional systems have been affected. Community 6 therefore reflects a knowledge organization pattern centered on brain functional mechanism analysis, underscoring the importance of combined fMRI and PET approaches in elucidating the relationship between functional brain activity and metabolic processes.

##### Community 7: FDG-PET Metabolic Imaging and Structural MRI Fusion Knowledge Cluster

Community 7 comprised 238 nodes and 310 internal connections, representing a knowledge cluster strongly oriented toward Alzheimer’s disease diagnosis. FDG-PET and structural MRI served as the principal hubs, with CSF markers and the anterior cingulate cortex constituting important secondary nodes. The community aggregated 25 Alzheimer’s disease-related nodes and 23 PET method nodes, reflecting a pronounced disease diagnosis-oriented character. Relationship analysis revealed that DIAGNOSTICALLY SYNERGIZES, SPATIALLY COMPLEMENTS, and CONVERGES WITH were the dominant association types, with diagnostically synergistic and spatial complementarity relations collectively forming the primary knowledge organization patterns within this community.

The extensive connections between CSF markers, FDG-PET, and structural MRI in the knowledge graph suggest that current research has progressively sought to establish systematic, multi-level associations among fluid biomarkers, brain metabolic abnormalities, and structural brain damage. When CSF biomarkers indicate that a pathological process has been initiated, FDG-PET can further delineate the extent of neuronal functional impairment, while structural MRI can capture the long-term structural consequences of disease progression. The integration of evidence across these complementary levels may therefore enhance the reliability of disease staging and progression assessment. A promising direction for future multimodal diagnostic research lies in the development of an integrated assessment framework incorporating fluid biomarkers, metabolic imaging, and structural imaging, with the aim of enabling early diagnosis, accurate disease staging, and progression prediction in Alzheimer’s disease.

### 3.5. Cross-Modal Consistency Analysis

**Figure 5:**
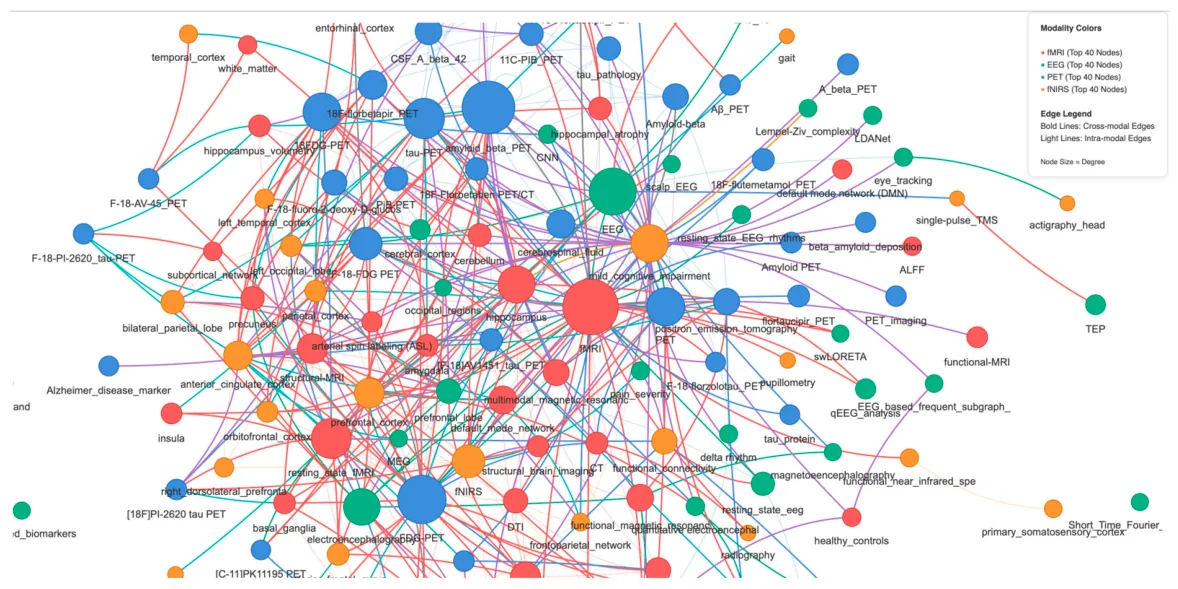
Cross-modal connection map of major modalities.

To further examine the association patterns among neuroimaging modalities, pairwise cross-modal association analysis was conducted for major modality combinations (Table 16). Cross-modal association was quantified using direct-edge density. Edge density was calculated as the number of direct cross-modal edges divided by the product of the numbers of nodes in the two modalities, reflecting the density of direct connections between modality-specific subgraphs. As shown in Figure 5, modality combinations varied substantially in terms of direct connection count, common neighbor size, and network embedding position. Some combinations formed dense cross-modal association networks, while others exhibited more localized complementarity relationships, reflecting heterogeneous degrees of synergy and complementarity among neuroimaging modalities at the level of information representation.

The fMRI-PET combination exhibited the largest number of direct cross-modal connections (401) and shared neighbors (189), with an edge density of 0.148%. These results indicate a substantial level of direct connectivity and knowledge overlap between fMRI and PET in the extracted knowledge network. DIAGNOSTICALLY SYNERGIZES was the dominant relation type, reflecting the frequent integration of functional and metabolic or molecular information in the literature. In individuals at the mild cognitive impairment (MCI) stage, PET may first indicate elevated Aβ or tau-related pathological burden, while fMRI can further reveal reduced functional connectivity within memory networks. Their combination may therefore facilitate the identification of high-risk individuals before structural atrophy becomes apparent, enabling a diagnostic shift from pathological change detection toward functional impairment capture at an earlier stage. The high-frequency synergistic relations characterizing this modality combination reflect not only the current research emphasis on multi-level brain information integration, but also suggest that future diagnostic frameworks for Alzheimer’s disease are likely to favor a comprehensive assessment approach combining molecular pathology and functional network evidence, with the aim of improving the accuracy of early identification and disease staging.

The fMRI-fNIRS combination comprised 107 direct connections and 120 common neighbor nodes, with an edge density of 0.281%. FUSES WITH and CONVERGES WITH were the dominant relation types. Despite differences in node scale, the relatively large number of common neighbor nodes indicates a high degree of consistency in research topics, disease scenarios, and analytical methods between the two modalities. From a diagnostic perspective, fMRI provides high spatial resolution information on brain functional localization, while fNIRS captures neural activity states by monitoring cerebral blood oxygen changes. As both modalities are grounded in neurovascular coupling mechanisms, they can characterize brain functional abnormalities from complementary physiological levels. Compared with fMRI, fNIRS offers practical advantages in terms of lower cost, greater portability, and less demanding acquisition requirements, making it particularly well suited for community screening, primary healthcare settings, and long-term follow-up scenarios. In summary, for conditions such as cognitive impairment and depressive disorder, fNIRS may serve as an accessible complement to high-cost MRI examinations, supporting early screening and dynamic monitoring and thereby broadening the coverage of brain function assessment in clinical practice.

The fNIRS-PET combination comprised 116 direct connections and 89 shared neighboring nodes, with an edge density of 0.283%. DIAGNOSTICALLY SYNERGIZES emerged as the dominant relation type. This pattern indicates a stable synergistic interaction between the two modalities in disease identification and computer-aided diagnosis research, reflecting their complementary roles in integrating pathological and functional evidence. From a diagnostic logic perspective, PET provides molecular-level pathological information, including amyloid-β deposition, tau pathology, and cerebral metabolic alterations, whereas fNIRS captures hemodynamic responses that reflect functional impairments induced by these underlying pathological changes. In the diagnosis of neurodegenerative disorders such as Alzheimer’s disease, this complementarity supports a joint diagnostic framework in which PET functions as a confirmatory tool for pathological abnormalities, while fNIRS serves as a complementary modality for functional assessment. For patients who are unable to undergo frequent PET examinations, fNIRS offers a feasible approach for longitudinal monitoring of cerebral functional dynamics, thereby enabling continuous evaluation of disease progression and supporting long-term clinical management. Moreover, with the advancement of portable and wearable fNIRS technologies, the fNIRS–PET pairing is increasingly positioned as a bridge between high-precision molecular imaging and routine functional monitoring. This integration provides a promising technical pathway for early detection, dynamic tracking, and long-term management of neurodegenerative diseases.

The fMRI-EEG combination comprised 102 direct connections and 97 common neighbor nodes, with an edge density of 0.045%. The two modalities exhibit clear complementarity at the level of information representation: the high temporal resolution of EEG compensates for the temporal limitations of fMRI, while the spatial localization precision of fMRI addresses the inherently limited spatial resolution of EEG. Their combined use therefore enables the simultaneous acquisition of complementary brain functional information across both temporal and spatial dimensions. In the knowledge graph, this complementarity is primarily reflected through FUSES WITH and SPATIALLY COMPLEMENTS relations, with the 97 common neighbor nodes further indicating extensive knowledge overlap between the two modalities in brain function research and disease mechanism investigation. In studies of depressive disorder, epilepsy, and cognitive impairment, the joint analysis of EEG and fMRI may support the development of more stable and objective brain functional abnormality markers, potentially improving both the accuracy and interpretability of disease identification.

The EEG-fNIRS combination comprised 69 direct connections and 61 common neighbor nodes, with an edge density of 0.200%. DIAGNOSTICALLY SYNERGIZES was the dominant relation type, indicating that existing research has focused considerably on exploring the combined application of these two modalities in disease identification and computer-aided diagnosis. EEG and fNIRS capture neuronal electrical activity at millisecond-level temporal resolution and monitor cerebral blood oxygen changes, forming complementary observation windows of the neurovascular coupling mechanism from electrophysiological and hemodynamic dimensions. Compared with large-scale equipment such as MRI and PET, both EEG and fNIRS offer advantages including non-invasiveness, portability, lower cost, and the capacity for repeated measurement, making them better suited for community screening, primary healthcare settings, and long-term follow-up scenarios. Therefore, for conditions such as Alzheimer’s disease, depressive disorder, and attention deficit hyperactivity disorder (ADHD), a lightweight computer-aided diagnostic system based on EEG–fNIRS fusion may improve early screening capacity and disease monitoring efficiency while maintaining low examination costs.

The EEG-PET combination comprised 42 direct connections and 104 common neighbor nodes, with an edge density of 0.017%. The number of common neighbor nodes substantially exceeded the number of direct connections, indicating considerable knowledge overlap between EEG and PET across disease research, brain region representation, and biomarker investigation. The dominant relation types were TEMPORALLY INFORMS and CONVERGES WITH, suggesting that the two modalities more commonly complement each other through evidence derived from distinct physiological levels. From a diagnostic perspective, PET is primarily employed to detect disease-related molecular pathological changes, including amyloid deposition, tau protein abnormalities, and brain metabolic alterations, thereby reflecting the pathological basis underlying disease onset and progression. EEG captures the functional impact of these pathological changes on neural network activity, reflecting dynamic abnormalities in brain information processing across dimensions such as frequency band power, functional connectivity, and cognitive evoked responses. The two modalities thus capture complementary pathological and functional dimensions of disease, forming a complementary relationship that spans from underlying causes to observable functional effects. Future research may therefore benefit from further integrating pathological imaging with neuroelectrophysiological information. For instance, in the diagnosis and stratified assessment of Alzheimer’s disease, PET could confirm the pathological deposition of amyloid-β or tau protein, while EEG simultaneously provides a dynamic assessment of neural network functional impairment, thereby supporting the construction of a cross-level multimodal diagnostic framework.

### 3.6. Candidate Biomarkers for Specific Diseases

**Figure 6:**
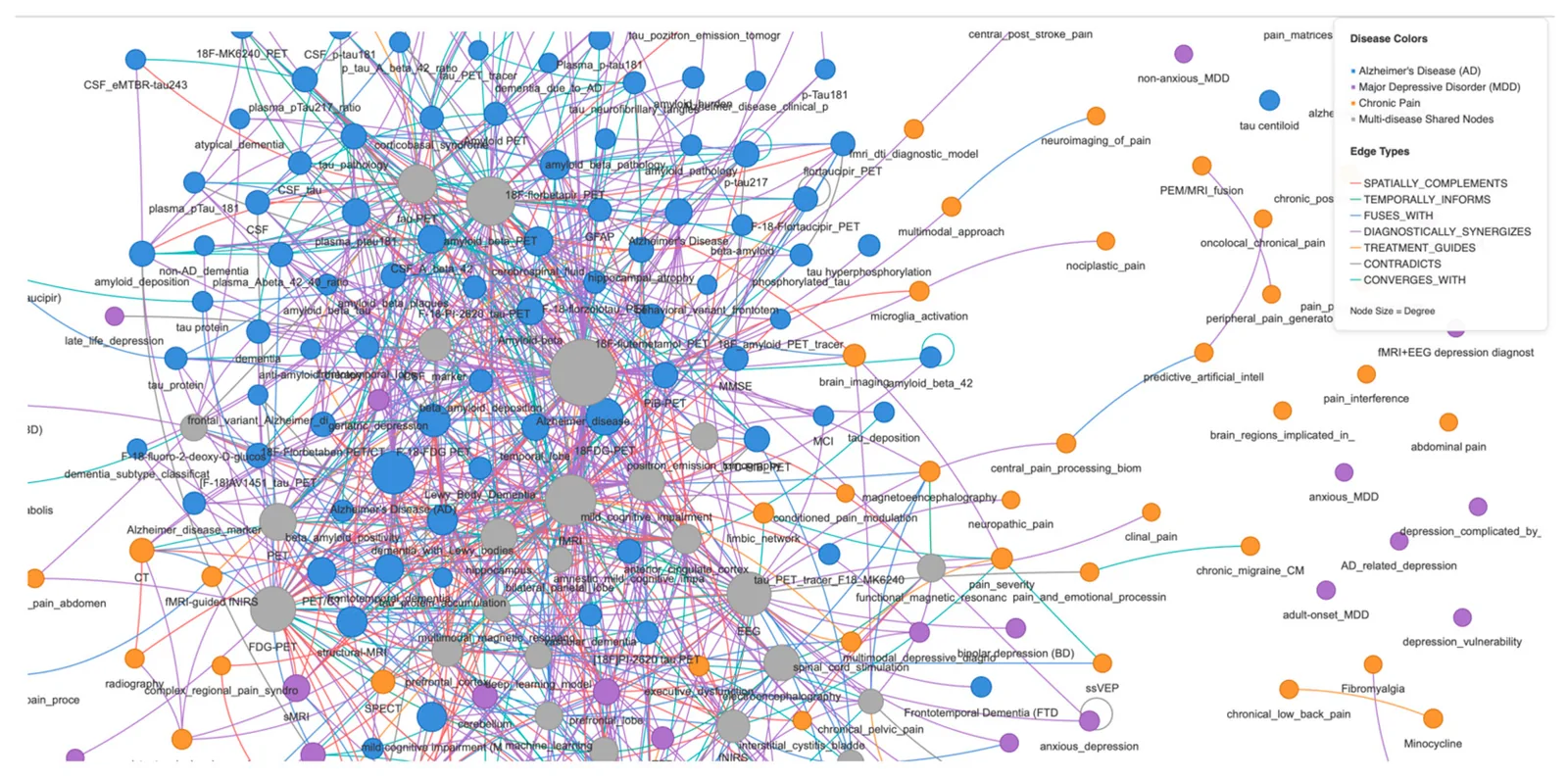
Disease subgraph.

To identify key biomarkers and their knowledge organization characteristics across different disease domains, disease-specific analyses were conducted centered on disease entity nodes (Figure 6). Major depressive disorder (MDD), Alzheimer’s disease (AD), and chronic pain were selected as seed nodes. First-order neighbor nodes maintaining direct connections to these seed nodes, together with their corresponding relation edges, were extracted to construct disease-specific knowledge subgraphs. Node connectivity degree within each subgraph was subsequently used to rank entities, and representative nodes with comparatively higher connectivity degrees were selected for further analysis (Table 17).

#### 3.6.1. Major Depressive Disorder

The MDD-centered subgraph exhibited pronounced multimodal organizational characteristics. High-connectivity nodes were primarily concentrated on major depressive disorder and its associated diagnostic entities, indicating that current research is largely oriented toward disease identification and diagnostic support [11,12]. Within the network, bipolar disorder emerged as the non-target disease with the highest connectivity, reflecting substantial overlap between the two conditions in symptomatology, neural mechanisms, and clinical differential diagnosis [13].

The core network also included subtype-specific entities such as geriatric depression, suggesting a gradual extension of neuroimaging research toward population-level and subtype-level stratification [14]. From a structural perspective, highly connected nodes were mainly organized around diagnostic labels, clinical phenotypes, and functional abnormalities, whereas molecular biomarker-level entities remained limited in centrality. Compared with neurodegenerative disorders such as Alzheimer’s disease, the MDD subgraph lacks a well-established biomarker-centered framework and is more dependent on system-level indicators, including functional connectivity patterns and large-scale brain network abnormalities [15,16]. Therefore, current MDD neuroimaging research remains centered on functional network alterations and multimodal feature integration for disease classification, while standardized biomarker frameworks are still under development [14,16].

#### 3.6.2. Alzheimer’s Disease

Compared with the MDD-centered subgraph, the AD-centered subgraph exhibited a more complete and hierarchically organized knowledge structure. The k(v) of the Alzheimer’s disease node reached 767, substantially exceeding that of other disease-related nodes, indicating that AD research within the multimodal neuroimaging field has developed a systematic framework encompassing disease states, pathological markers, fluid biomarkers, and prodromal clinical stages.

At the pathological marker level, amyloid-β PET and tau-PET exhibited k(v) of 320 and 156, respectively, representing the most central entities after the disease node itself. These two modalities capture the pathological progression of AD through amyloid deposition and neurofibrillary tangle formation, respectively, and constitute the primary sources of evidence in current research on pathological diagnosis [15,17]. Fluid biomarkers such as CSF Aβ42, despite their relatively lower connectivity degrees, provide complementary assessments of disease progression from both fluid and imaging perspectives, collectively supporting pathological staging and computer-aided diagnosis. The high centrality of these markers in the knowledge graph indicates that molecular imaging of pathological protein deposition has become a core focus of current research on early identification, pathological subtyping, and computer-aided diagnosis [18]. At the prodromal stage of AD, the MCI node achieved a k(v) of 124, serving as an important hub connecting risk assessment, early identification, and disease progression prediction. In the knowledge network, MCI is associated with multiple research directions, including early screening, disease conversion prediction, and intervention timing assessment. A substantial body of research has employed PET imaging, fluid biomarkers, and multimodal neuroimaging features to identify individuals at high risk of conversion from MCI to AD, reflecting a gradual shift in research focus from disease confirmation toward ultra-early detection and risk stratification [19,20].

Therefore, the AD-centered subgraph encompasses knowledge entities across multiple levels, including disease states, molecular imaging markers, fluid biomarkers, and prodromal clinical stages, forming a relatively complete and hierarchically organized evidence structure. Compared with the MDD and chronic pain subgraphs, the candidate biomarker framework in the AD field is more extensive and the knowledge structure more concentrated, reflecting the emergence of a comparatively mature research framework underpinned by a stable and well-established evidence base [14,19].

#### 3.6.3. Candidate Biomarkers for Chronic Pain

Compared with the AD and MDD subgraphs, the chronic pain-centered subgraph was smaller in overall scale, with generally lower node connectivity degrees, and had not yet formed a core biomarker framework with prominent centrality. In terms of node composition, the subgraph primarily comprised entities such as pain severity, central pain processing biomarkers, and pain-emotional processing circuits, encompassing brain functional abnormalities related to pain perception, pain processing, and emotional regulation. Unlike Alzheimer’s disease, the characterization of chronic pain is more strongly grounded in abnormal brain functional network activity and alterations in pain processing mechanisms, reflecting pronounced system-level functional characteristics [21,22]. Pain severity has been primarily assessed using EEG-derived measures, whereas chronic pain diagnostic markers were more closely associated with the fNIRS modality, reflecting current research efforts to objectively quantify pain states from both neuroelectrical and cerebral hemodynamic perspectives [23].

The clinical diagnosis and treatment evaluation of chronic pain have long relied predominantly on patients’ subjective symptom reports and scale-based assessments, with a corresponding lack of stable and reliable objective indicators [24]. The incorporation of neuroimaging evidence therefore offers a promising research pathway toward the construction of an objective pain assessment framework [25]. The knowledge graph findings suggest that an important direction for future chronic pain research may lie in the integration of multidimensional brain functional information derived from EEG, fNIRS, and other neuroimaging modalities, with the aim of establishing a pain assessment framework grounded in objective neurophysiological features. Quantifying abnormal pain-related brain network patterns may not only improve the accuracy of disease identification and stratified management, but also provide more objective criteria for treatment monitoring and individualized intervention, thereby advancing a transition in chronic pain diagnosis and management from subjective symptom assessment toward objective biomarker-assisted clinical decision-making.

External clinical and biological evidence further supports the observed differences in biomarker organization across diseases. For Alzheimer’s disease (AD), biomarker-based assessment has achieved substantial progress toward clinical translation. In 2025, the U.S. Food and Drug Administration (FDA) cleared the Lumipulse G pTau217/β-Amyloid 1–42 Plasma Ratio as the first blood-based in vitro diagnostic device authorized to aid in the diagnosis of AD. In a multicenter study of 499 cognitively impaired individuals, positive and negative test results showed 91.7% and 97.3% agreement, respectively, with amyloid pathology established by amyloid positron emission tomography (PET) or cerebrospinal fluid (CSF) testing (FDA, 2025) [26]. This development is also consistent with the Alzheimer’s Association Clinical Practice Guideline, which provides evidence-based recommendations for the clinical use of blood-based biomarkers, with CSF biomarkers, amyloid PET, or neuropathological assessment serving as reference standards (Palmqvist et al., 2025) [27]. In contrast, although numerous candidate biomarkers have been investigated for major depressive disorder (MDD), their diagnostic validity and clinical utility remain insufficiently established (Ang et al., 2025) [28]. Similarly, neuroimaging biomarkers for chronic pain continue to face challenges in validation, clinical utility, and generalizability (Mackey et al., 2019; Davis et al., 2017) [29,30]. Collectively, these independent clinical and biological findings are broadly consistent with the relative differences in biomarker maturity identified in the knowledge graph and provide external support for the comparatively more established and systematically organized biomarker framework observed in AD than in MDD and chronic pain.

## 4. Discussion

### 4.1. Interpretation of Core Findings

The neuroimaging knowledge graph constructed in this study using LLMs reveals the underlying knowledge organization structure and core research foci within current multimodal fusion research. The identified disease hubs, brain region hubs, and cross-modal bridging nodes collectively reflect the distribution and accumulation of knowledge within this domain.

At the disease level, Alzheimer’s disease demonstrates a particularly pronounced concentration of knowledge. Grounded in the AT(N) biomarker framework, multimodal evidence from PET, MRI, cerebrospinal fluid (CSF) assays, and blood-based biomarkers has progressively converged on core pathological processes, including amyloid-β deposition, tau pathology, and neurodegeneration [31]. This convergence has led to the formation of a relatively mature framework for cross-modal evidence integration in the field, which is reflected in the high centrality of Alzheimer’s disease–related nodes within the knowledge graph. This structural pattern carries important implications for both biomedical research and clinical diagnostic practice.

For medical research, future Alzheimer’s disease studies should move toward more fine-grained integration of cross-modal evidence, systematically evaluating which combinations of modalities yield the strongest joint diagnostic performance across different disease stages, including the preclinical, mild cognitive impairment (MCI), and dementia phases. Particular attention should be given to whether the coupling strength among Aβ-PET, tau-PET, and resting-state fMRI-derived functional connectivity indices varies as the disease progresses. From a clinical diagnostic perspective, Alzheimer’s disease has now established a relatively complete multimodal evidence chain encompassing Aβ-PET, tau-PET, cerebrospinal fluid (CSF) biomarkers, and functional MRI connectivity measures. The high-density DIAGNOSTICALLY SYNERGIZES relationships among these modalities in the knowledge graph further support a stratified diagnostic framework in clinical practice. In this framework, blood-based p-tau217 can serve as a low-cost initial screening tool; individuals with positive results would then undergo Aβ-PET to confirm pathological burden, followed by resting-state fMRI to assess the extent of functional network disruption. This three-tiered sequential evidence structure enhances the feasibility of early detection and supports a more structured approach to disease staging and monitoring [15,32].

In contrast, the knowledge structure of major depressive disorder (MDD) exhibits pronounced dispersion [13]. Existing research evidence is distributed across multiple explanatory frameworks, including brain network abnormalities, emotional regulation dysfunction, and neurotransmitter alterations, without a unified pathological anchor [33]. This is reflected in the knowledge graph as scattered connectivity patterns and the absence of dominant core hubs, suggesting that a coherent biomarker framework in this field remains underdeveloped. For MDD, it is therefore necessary to construct a stratified biomarker framework inspired by the design logic of the AT(N) system. Such a framework could be organized around relatively stable neurobiological features, such as default mode network dysregulation and abnormalities in prefrontal–limbic circuitry [34], with the goal of transforming fragmented descriptive findings into a more systematic and integrative knowledge structure. From a clinical diagnostic perspective, a practical strategy at the current stage is to combine portable neuroimaging technologies, such as EEG and fNIRS, as auxiliary screening tools. Their complementary strengths in assessing prefrontal functional dynamics may provide preliminary neurophysiological evidence for early evaluation. Resting-state fMRI can then be incorporated for further stratification and subtype identification when conditions permit, enabling differentiation of neurobiological subtypes and supporting more individualized treatment decisions.

At the brain region level, the hippocampus, prefrontal cortex, and anterior cingulate cortex emerged as core neuroanatomical hubs within the knowledge graph. Among these, the hippocampus, recognized as a primary site of early pathological alteration in Alzheimer’s disease [15], exhibits particularly strong cross-modal convergence. Structural MRI, functional MRI, and PET jointly characterize the hippocampus from complementary dimensions, capturing structural atrophy, functional connectivity disruptions, and pathological protein deposition. The convergence of these heterogeneous evidence sources onto a single anatomical node positions the hippocampus as one of the most robustly supported neuroanatomical targets in current multimodal research. When structural atrophy, reduced functional connectivity, and Aβ/tau pathological burden co-occur within the same individual, cross-modal consistency provides mutually reinforcing diagnostic evidence. This integrative validation can enhance reliability in early-stage identification, even prior to pronounced macroscopic atrophy, thereby potentially extending the window for early intervention.

The prefrontal cortex and anterior cingulate cortex also exhibit high node centrality within the knowledge graph, with extensive connections spanning multiple disease domains, including Alzheimer’s disease, major depressive disorder, attention deficit hyperactivity disorder (ADHD), and chronic pain. This cross-disease co-occurrence pattern suggests that these regions play a central role across a range of neuropsychiatric conditions, where functional abnormalities may reflect a shared disruption of cognitive control and emotional regulation networks across distinct pathological contexts [35,36]. From a research perspective, this provides a consensus-driven entry point for cross-disease comparative neuroimaging studies, facilitating the identification of both shared mechanisms and disorder-specific characteristics within cognitive control and affective regulation systems.

Beyond disease and brain region nodes, the bridging nodes within the graph further reveal the structural characteristics of multimodal knowledge integration. Fluid biomarkers, such as cerebrospinal fluid (CSF) Aβ42, are simultaneously connected to multiple neuroimaging modalities, functioning as mediators between molecular pathological information and imaging-based evidence [37]. In this role, they establish explicit links within the knowledge network between fluid biomarker findings and imaging results derived from PET and MRI. Given their relatively low collection cost and suitability for repeated measurements, fluid biomarkers are well positioned as primary entry points for large-scale screening. Their extensive cross-modal connectivity within the graph further suggests a shared conceptual and methodological foundation for constructing a stratified “screening–confirmation” diagnostic pathway in clinical practice. The Machine Learning node also demonstrates strong bridging characteristics, with connections spanning multiple modalities, including fMRI, EEG, PET, and fNIRS. This pattern reflects the broad cross-modal adaptability of machine learning methods in handling heterogeneous, high-dimensional neuroimaging data [38].

### 4.2. Comparison with Existing Manual Reviews and Databases

Knowledge organization in the neuroimaging field has traditionally relied on two main approaches: manual literature reviews and specialized neuroimaging databases. Manual reviews offer in-depth theoretical interpretation and mechanistic insights into specific diseases or research topics; however, the process of knowledge integration is heavily dependent on researchers’ ability to select, interpret, and synthesize the literature. As the volume of neuroimaging publications continues to expand across multiple modalities, it has become increasingly challenging to systematically track the complex relational structures among studies, particularly in capturing synergistic, complementary, or even conflicting relationships in cross-modal evidence [39]. Neuroimaging databases such as BrainMap and Neurosynth provide an important infrastructure for cross-study comparison and meta-analysis by aggregating large-scale activation coordinate data. Nevertheless, these platforms primarily organize knowledge around brain region activations and their spatial distributions, emphasizing statistical patterns of functional co-occurrence. As a result, they offer relatively limited representation of multimodal fusion relationships and higher-level semantic associations across different imaging modalities [40,41].

In this context, the knowledge graph constructed using large language models in this study offers a framework distinct from traditional approaches to knowledge organization. Rather than relying on manual curation or voxel-wise activation coordinates, the proposed framework standardizes heterogeneous literature into semantically normalized entities and typed, literature-weighted relations, enabling knowledge that was previously fragmented across modality-specific studies to be integrated into a unified, computable, and queryable network [42].

In terms of knowledge representation, this study differs fundamentally from existing neuroimaging databases such as BrainMap and Neurosynth. These platforms primarily rely on voxel-wise activation coordinates or brain region co-activation patterns as basic knowledge units, integrating activation information from large-scale neuroimaging studies to support spatial meta-analyses and cross-study functional association inference [40,41]. In contrast, the present study adopts entities and their semantic relations as the fundamental units of representation, explicitly encoding inter-modal relationships within a structured network. These relations include diagnostic synergy, information fusion, spatial complementarity, temporal information transfer, and evidence conflict. Under this representation, associations between concepts are characterized not only by the existence of a connection but also by its semantic type. For instance, the relationship between fMRI and EEG is primarily represented as spatiotemporal information complementarity, whereas the relationship between PET and fMRI is more strongly associated with diagnostic synergy and spatial complementarity. This relation-aware representation enables a more nuanced characterization of cross-modal interactions beyond conventional co-activation-based frameworks [43].

In terms of modality coverage, the constructed graph integrates multiple heterogeneous data sources, including fMRI, EEG, PET, fNIRS, MRI, DTI, MEG, as well as cerebrospinal fluid and blood biomarkers, and identifies a substantial number of cross-modal relational links. These findings suggest that core knowledge in the neuroimaging domain is progressively formed through cross-modal validation and information complementarity [44]. Accordingly, single-modality databases or reviews centered on a specific imaging technique are inherently limited in their ability to capture the global association structure across diverse evidence sources. In contrast, the proposed knowledge graph represents both the interconnections and organizational patterns among heterogeneous modalities within a unified network framework.

At the disease level, the constructed knowledge network spans multiple research domains, including Alzheimer’s disease, major depressive disorder, chronic pain, Parkinson’s disease, epilepsy, and anxiety disorders. Within a unified representation framework, the graph integrates shared brain regions, biomarkers, and analytical methods across these conditions, thereby enabling systematic cross-disease comparative analysis [45,46]. For instance, the anterior cingulate cortex is concurrently linked to studies of pain processing, emotional regulation, and cognitive control, while the hippocampus is broadly associated with memory function and neurodegenerative disease research [15,35]. These cross-disease connections are relatively densely distributed within the network, reflecting substantial overlap in research foci across disorders at the level of key neuroanatomical structures.

In addition, this study identified instances of conflicting evidence across different studies, suggesting that a degree of divergence in conclusions persists within current neuroimaging research. Traditional narrative reviews typically resolve such discrepancies through subjective synthesis by authors; in contrast, the proposed knowledge graph preserves contradictory findings in a structured form within a unified network, thereby treating evidence conflict itself as an analyzable object. This capability provides a new foundation for subsequent evidence quality assessment, methodological comparison, and systematic tracing of disputed findings, and represents a key advantage of the knowledge graph approach over conventional methods of knowledge organization.

### 4.3. Clinical Translation Implications

Classifying the evidence in the knowledge graph into three clinical translation stages, namely diagnosis, treatment, and prognosis, reveals a marked imbalance in knowledge distribution across these stages (Table 18). Diagnostically synergistic relations account for 41.1% of all connections, whereas evidence related to treatment guidance and prognosis prediction accounts for only 3.2% and 0.2%, respectively. This pattern indicates that current multimodal neuroimaging research is primarily concentrated on disease identification, while relatively limited progress has been made in linking imaging findings to treatment decision-making and outcome prediction across the clinical continuum.

**Figure 7:**
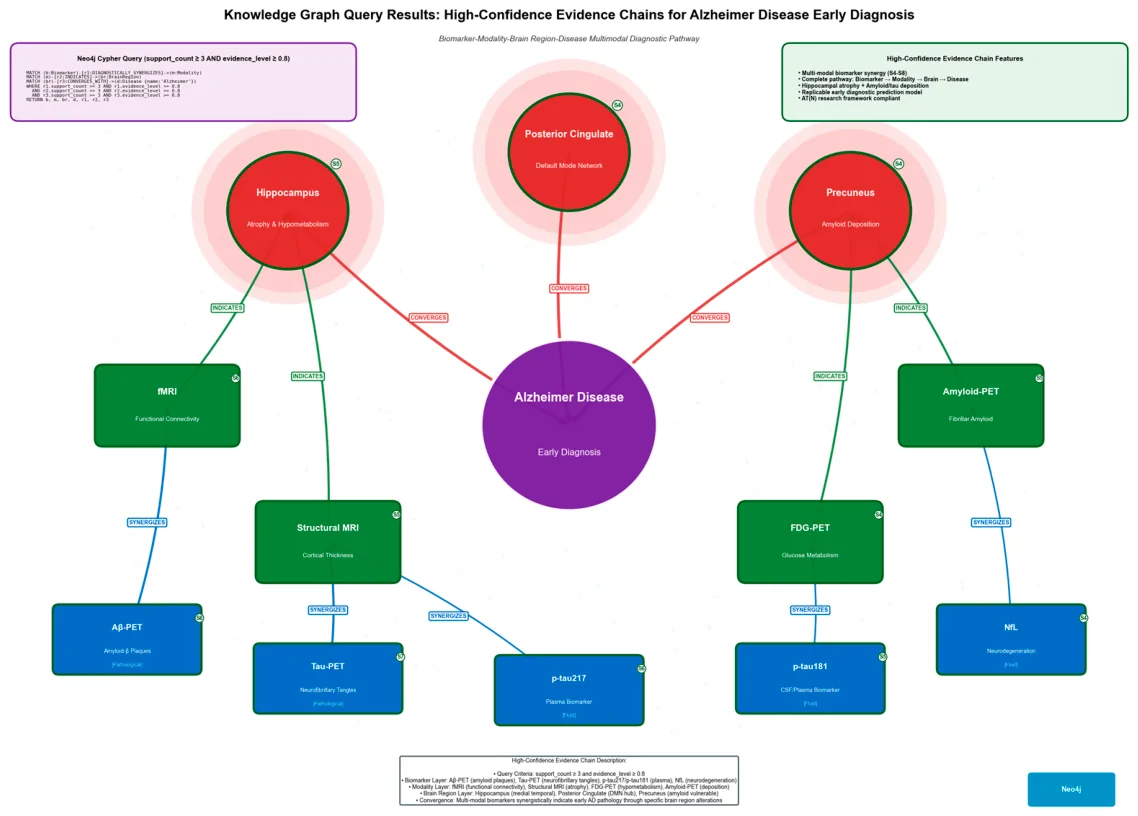
Evidence chain for early diagnosis of Alzheimer’s disease. Color coding: blue nodes represent biomarkers; red nodes denote brain regions; green nodes indicate neuroimaging modalities; purple node corresponds to Alzheimer disease (disease outcome). Arrow colors represent different semantic relations: blue for DIAGNOSTICALLY SYNERGIZES, green for INDICATES, red for CONVERGES. Badge “S” denotes literature-support count.

At the diagnostic stage, the knowledge graph provides clinicians with evidence-based references for constructing multimodal diagnostic pathways. Taking Alzheimer’s disease as an example, the graph contains a large number of diagnostically synergistic relations among amyloid-PET, tau-PET, resting-state fMRI functional connectivity, and cerebrospinal fluid biomarkers (Figure 7). These relations support a stratified diagnostic strategy in which fluid biomarkers are used for initial screening, PET is applied for pathological confirmation, and fMRI is used for functional assessment. This sequence allows clinicians to select modality combinations according to practical constraints, including test availability, cost considerations, and disease stage [15,32]. In primary healthcare settings, the diagnostically synergistic evidence associated with the EEG and fNIRS combination suggests that portable dual-modality screening may serve as an alternative when MRI and PET are not accessible. This approach can provide preliminary neuroimaging evidence while maintaining relatively low examination costs [47].

**Figure 8:**
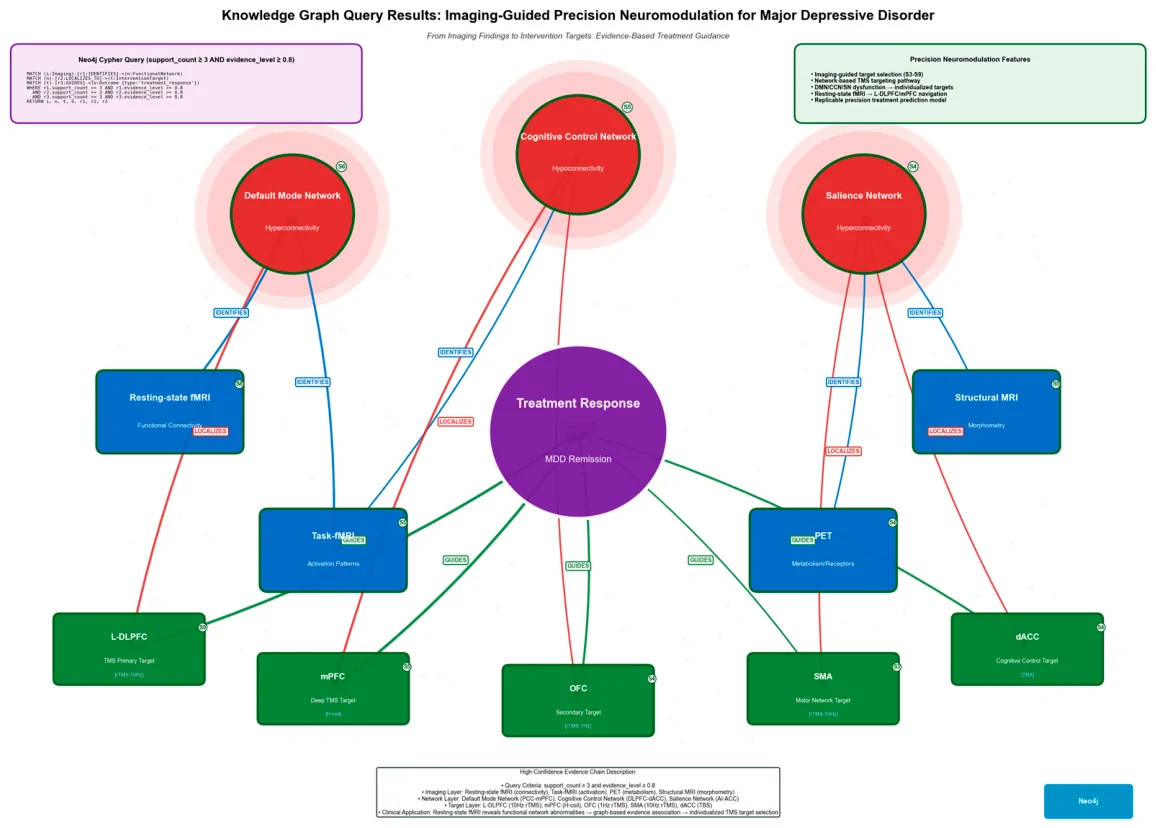
Knowledge graph of major depressive disorder. Color coding: blue nodes represent neuroimaging modalities; red nodes denote functional brain networks; green nodes indicate intervention targets; purple node corresponds to treatment outcome. Arrow colors represent different semantic relations: blue for IDENTIFIES, red for LOCALIZES, green for GUIDES. Badge “S” denotes literature-support count.

At the treatment guidance stage, the knowledge graph establishes preliminary links between imaging findings and therapeutic targets. For example, in major depressive disorder, direct connections between resting-state fMRI functional connectivity abnormalities and prefrontal transcranial magnetic stimulation (TMS) targets provide literature-based support for imaging-guided neuromodulation (Figure 8). By combining patient-specific resting-state fMRI findings with the evidence associations represented in the graph, clinicians may be assisted in identifying individualized TMS targets [48,49]. Similarly, the associations between PET imaging biomarkers and anti-amyloid therapies are consistent with current clinical trial practice, in which imaging biomarkers are used for patient selection and treatment response monitoring in Alzheimer’s disease [50].

In addition, fNIRS may enhance the clinical accessibility of neuroimaging-based assessment. In the knowledge graph, fNIRS shows direct associations with the prefrontal cortex, as well as relationships of spatial complementarity and diagnostic synergy with fMRI. These patterns suggest partial overlap between cortical hemodynamic signals measured by fNIRS and functional connectivity features derived from fMRI in disease characterization. Compared with PET and fMRI, fNIRS is more portable, lower in cost, and supports measurements in ecologically valid or ambulatory settings, which makes it more feasible for use in primary care for screening and longitudinal monitoring [47]. In settings where access to large-scale imaging infrastructure is limited, fNIRS may complement or partially substitute fMRI, particularly when combined with EEG in multimodal protocols. The associations captured in the knowledge graph provide structured literature support for such clinically oriented deployment scenarios.

### 4.4. Limitations and Future Directions

Despite the scale and breadth of the multimodal neuroimaging knowledge graph constructed in this study, which encompasses 1838 articles, 4190 entity nodes, and 7007 semantic relations, several limitations warrant consideration.

First, the cross-modal relations identified in the graph primarily represent evidence reported in the literature rather than causal or clinically validated relationships. Relations such as “diagnostic synergy,” “spatial complementarity,” and “treatment guidance” should therefore be interpreted as evidence-derived associations rather than direct indicators of clinical efficacy or predictive performance. Future work should empirically validate the findings associated with high-centrality nodes and key cross-modal associations using independent neuroimaging datasets, such as ADNI, UK Biobank, and the ABCD Study, to assess their consistency with empirical observations.

Second, although LLM-based extraction improves efficiency, its accuracy remains sensitive to terminology, reporting conventions, and source-text structure, potentially causing omissions or misclassification of negative findings, conflicting evidence, effect sizes, and study-quality information not explicitly represented in the current schema. Future work should adopt an iterative expert-guided refinement process to identify errors in entity recognition and normalization, relation identification, and semantic type assignment, and use these error patterns to refine the extraction schema, relation definitions, normalization rules, and output constraints. The resulting improvements can be quantified by comparing precision and Cohen’s κ before and after refinement, together with changes in unsupported nodes, relations, and semantic types. Controlled ablation experiments could further quantify the contributions of RAG-based retrieval, semantic merging, and network analysis to extraction accuracy and graph-level reliability.

Third, the framework also has potential for application beyond the current multimodal neuroimaging domain. Such transfer would primarily require adaptation of domain-specific search terms, extraction schemas, ontology mappings, and semantic validation rules, while retaining the core procedures for LLM-based extraction, post-processing, threshold-based filtering, and knowledge graph construction. Cross-domain generalizability should be evaluated using independent biomedical or neuroimaging datasets by comparing node-level and relation-level precision, together with graph-level consistency and reliability.

Finally, computational scalability and long-term maintenance should be considered for the knowledge graph. The complete processing workflow was conducted using a local computing environment with 48 GB of available memory, with the pipeline implemented in Python 3.9. LLM-based extraction of 1838 articles required approximately 18 h 11 min, corresponding to an average processing time of approximately 35.6 s per article. As articles were processed independently through API calls, the computational cost of the extraction stage scales approximately linearly with corpus size and represents the primary computational burden of the pipeline. Peak memory usage during the complete processing workflow was approximately 1.46 GB.

Regarding long-term maintenance, newly published literature can be incorporated incrementally. New records would undergo duplicate removal and the same relevance screening and structured extraction procedures used for the initial corpus. Newly extracted entities would then be compared with existing nodes using the established semantic similarity criterion; entity pairs with a similarity score of ≥0.85 would be considered for merging according to the predefined normalization rules, whereas those below the threshold would be retained as distinct candidates for further validation. Newly extracted relations would subsequently be matched against existing edges to identify new, repeated, or potentially conflicting associations. Only newly identified or modified nodes and relations would then be incorporated into the existing graph and subjected to the same threshold-based filtering and quality-control procedures. This strategy would minimize redundant LLM processing and graph reconstruction while enabling continuous incorporation of newly published neuroimaging evidence.

## 5. Conclusions

This study constructed a multimodal neuroimaging knowledge graph comprising 4190 entity nodes and 7007 semantic relations, derived from 1838 neuroimaging-related publications. The resulting graph integrates associations among neuroimaging modalities, diseases, biomarkers, brain regions, and analytical methods within a unified structured framework. Unlike conventional knowledge organization approaches based on manual curation or single-source databases, the proposed pipeline leverages large language models and retrieval-augmented generation for entity recognition, relation extraction, and knowledge fusion, enabling scalable construction of structured knowledge from heterogeneous neuroimaging literature.

Graph structure analysis revealed pronounced multimodal integration in current neuroimaging research, with cross-modal relations accounting for 63.2% of all edges. Diagnostically synergistic associations were the most prevalent relation type, whereas evidence pertaining to treatment guidance and prognosis prediction remained comparatively sparse. Community detection further showed that Alzheimer’s disease-related studies form multiple high-density communities, reflecting a relatively mature and well-structured knowledge organization within multimodal neuroimaging, whereas research on major depressive disorder is organized predominantly around resting-state functional connectivity and brain network abnormalities, with a narrower methodological and biomarker distribution. Cross-modal consistency analysis indicated that fMRI occupies a central bridging position within the network, exhibiting substantial overlap with PET, EEG, and fNIRS; the fMRI–PET pairing showed the strongest cross-modal association, whereas fMRI–EEG and fMRI–fNIRS primarily reflected complementary sensitivity to electrophysiological and hemodynamic processes. Disease-specific subgraphs further revealed marked variability in evidence maturity: Alzheimer’s disease exhibited dense multimodal convergence onto a stable biomarker set, whereas major depressive disorder and chronic pain remained characterized by more heterogeneous and less centralized knowledge structures.

Collectively, these findings indicate that current knowledge accumulation in multimodal neuroimaging is heavily concentrated on diagnostic applications, while evidence supporting treatment guidance and prognosis prediction remains comparatively underdeveloped, and that neurodegenerative and psychiatric or pain-related conditions differ markedly in the maturity of their underlying evidence base. Future work should therefore prioritize expanding literature coverage for underrepresented disease domains, incorporating external validation against open neuroimaging datasets, and integrating human-in-the-loop verification to enhance the robustness of the extraction pipeline. More broadly, the proposed LLM-augmented knowledge graph framework constitutes a scalable approach for organizing and analyzing multimodal neuroimaging literature, providing a structured foundation for future evidence integration and biomarker discovery.

## Figures and Tables

**Table 1:** Literature search terms.

Theme Group	Search Terms
Imaging Technique-related Terms	functional magnetic resonance imaging; fMRI; functional MRI; BOLD; blood oxygenation level dependent; electroencephalography; EEG; electroencephalogram; event-related potential; ERP; functional near-infrared spectroscopy; fNIRS; fNIR; NIRS; near-infrared spectroscopy; hemoglobin concentration; oxyhemoglobin; deoxyhemoglobin; diffuse optical tomography; positron emission tomography; PET; PET imaging; PET/CT; PET/MRI; FDG-PET, etc.
Brain Activation-related Terms	brain activation; brain region; neural activity; brain mapping; cortical activation; activation pattern; functional activation; regional activation; localized activation; hemodynamic response; BOLD signal; neural activation; brain area activation; activation foci; activation cluster; activation map; spatial pattern; temporal pattern; network activation; co-activation; deactivation
Experimental Task-related Terms	paradigm; cognitive task; experimental task; task design; task condition; task performance; behavioral task; functional task; stimulus presentation; block design; event-related design; task switching; working memory task; attention task; language task; motor task; visual task; auditory task; memory task; executive function task; resting state; control task; baseline condition

**Table 2:** Literature search workflow.

Database	Records Identified
PubMed, Web of Science, Scopus, and PsycINFO	14,891
Additional records identified through reference tracking	6081
Records after merging	20,972
Records retained after PRISMA screening	1838

**Table 3:** Prompt engineering components.

Module	Functional Description
Role Definition	Defines the model as an expert in neuroimaging knowledge graph construction
Domain Knowledge Injection	Injects foundational knowledge on multimodal neuroimaging technologies and their clinical applications
Extraction Focus	Emphasizes key knowledge categories, including spatial complementarity, temporal complementarity, diagnostic synergy, and treatment guidance
Schema Constraints	Restricts the extraction scope to predefined entity types and relation types
Output Constraints	Enforces a standardized JSON-format output structure

**Table 4:** Definitions of entity types.

Category	Description
Neuroimaging Modality	Neuroimaging techniques such as fMRI, EEG, fNIRS, PET, etc.
Brain Region	Functional brain areas including the hippocampus, prefrontal cortex, amygdala, etc.
Clinical Condition	Disease states such as Alzheimer’s disease, depression, Parkinson’s disease, etc.
Diagnostic Marker	Multimodal diagnostic biomarkers
Treatment Target	Therapeutic targets such as TMS targets, DBS targets, etc.
Fusion Method	Fusion methods such as EEG-informed fMRI, fMRI-guided fNIRS, etc.

**Table 5:** Definitions of relation types.

Relation Type	Semantic Definition
SPATIALLY COMPLEMENTS	Spatial information complementarity
TEMPORALLY INFORMS	Temporal information complementarity
FUSES WITH	Technical fusion relationship
DIAGNOSTICALLY SYNERGIZES	Diagnostic synergistic relationship
TREATMENT GUIDES	Treatment guidance relationship
CONVERGES WITH	Cross-modal convergence relationship
CONTRADICTS	Cross-modal contradictory relationship

**Table 6:** Structured Prompt Framework for LLM-Based Knowledge Extraction.

Constraint	Content
Role Definition	The model is instructed to perform structured knowledge extraction from multimodal neuroimaging literature, with emphasis on fMRI, EEG, fNIRS, and PET.
Step-0 Language Rules	Entity names, relation types, and attributes follow predefined naming conventions. Standard abbreviations (e.g., fMRI, EEG, fNIRS, and PET) are retained where appropriate. Predefined entity categories include neuroimaging modality, brain region, clinical condition, diagnostic marker, treatment target, and fusion method.
Step-1 Semantic Merging	Predefined relations include SPATIALLY COMPLEMENTS, TEMPORALLY INFORMS, FUSES WITH, DIAGNOSTICALLY SYNERGIZES, TREATMENT GUIDES, CONVERGES WITH, and CONTRADICTS.
Step-2 Entity Type Definitions	Synonymous or semantically equivalent entities are normalized according to predefined synonym-mapping and semantic-similarity rules, with a similarity threshold of 0.85.
Step-3 Relation Types and Directionality	Extracted knowledge units are retained according to predefined evidence-support and frequency-based filtering criteria.
Step-4 Quantity and Quality Constraints	Structured JSON output is required, including entity IDs, entity types, relations, and literature-support information.
Step-5 Output Format	Language checking → entity extraction → relation extraction → evidence assessment → filtering → structured output.

**Table 7:** Manual validation of LLM-based knowledge extraction.

Validation Item	Num	Validation Item	Num	Validation Item	Num
Documents reviewed	50	Correctly identified nodes	127	Correctly identified relations	67
Nodes evaluated	144	Extraction precision (nodes)	88.2%	Extraction precision (relations)	85.6%
Relations evaluated	78				
Cohen’s κ	0.76				

**Table 8:** Frequency Distribution of Candidate Knowledge Nodes and Relations.

Occurrence Frequency	Node Count	Proportion	Relation Count	Proportion
1	12,719	57.94%	10,252	59.40%
2	5042	22.97%	3431	19.88%
3	2191	9.98%	1536	8.90%
4	900	4.10%	734	4.25%
5	470	2.14%	389	2.25%
6–9	514	2.34%	704	4.08%
≥10	115	0.52%	213	1.23%
Raw Candidate Nodes/Relations	21,951	100.00%	17,259	100.00%

**Table 9:** Comparison of knowledge extraction scale before and after pruning.

Metric	Raw Extraction	After Pruning	Retention Rate
Nodes	21,951	4190	19.09%
Relations	17,259	7007	40.60%
Processed Documents	1823		

**Table 10:** Frequency statistics of main entities and relations.

Relation Type	Count (%)	Entity Type	Count (%)
DIAGNOSTICALLY SYNERGIZES	2881 (41.10%)	diagnostic marker	1412 (33.70%)
FUSES WITH	1464 (20.90%)	brain region	1008 (24.10%)
CONVERGES WITH	978 (14.00%)	fusion method	945 (22.60%)
SPATIALLY COMPLEMENTS	968 (13.80%)	modality	644 (15.40%)
CONTRADICTS	344 (4.90%)	treatment target	144 (3.40%)
TREATMENT GUIDES	226 (3.20%)		
TEMPORALLY INFORMS	144 (2.10%)		

**Table 11:** Knowledge graph Topological Feature statistics.

	Metric	Value
Overall graph	Total Nodes	4190
	Total Relations	7007
	Overall Graph Density	3.99 × 10^−4^
	Total Connected Components	783
Connected components	Single-node Isolated Components	629
	Two-node Components	108
Largest WCC	Maximum WCC Nodes	3209
	WCC Coverage Rate	76.60%
	WCC Density	6.54 × 10^−4^
	WCC Average Degree	4.20
	Internal WCC Edges	6736
	WCC Edge Proportion	96.10%
	Average External WCC Degree	0.51
Largest SCC	Maximum SCC Nodes	641
	SCC Coverage Rate	15.30%

**Table 12:** Hub nodes.

Node	Degree	Betweenness	Eigenvector Centrality	Closeness Centrality
Alzheimer’s disease	767	0.2267	0.1461	0.1057
fMRI	366	0.1037	0.0867	0.1029
mild cognitive impairment	124	0.0192	0.0474	0.0945
EEG	236	0.0772	0.0168	0.0874
amyloid-β PET	320	0.0649	0.0623	0.0813
FDG-PET	256	0.0515	0.0231	0.0992
major depressive disorder	198	0.0488	0.0742	0.098
resting-state fMRI	151	0.0264	0.1122	0.1053

**Table 13:** Topological properties of modality-specific subgraphs.

Modality	Nodes	Edges	Density	Clustering Coefficient
multimodal	2126	1288	0.0003	0.1824
PET	540	464	0.0016	0.2209
fMRI	501	432	0.0017	0.2947
EEG	454	204	0.001	0.0281
fNIRS	76	60	0.0105	0.4135

**Table 14:** Core brain regions.

Brain Region Node	Degree	Cross-Modal Edges	Dominant Relation Type (Num)
hippocampus	121	68	SPATIALLY COMPLEMENTS (46)
prefrontal cortex	63	23	SPATIALLY COMPLEMENTS (24)
anterior cingulate cortex	56	24	CONVERGES WITH (21)
default mode network	50	20	CONVERGES WITH (18)
temporal lobe	37	16	SPATIALLY COMPLEMENTS (15)
amygdala	20	5	CONVERGES WITH (9)
thalamus	22	6	CONVERGES WITH (8)
insula	16	3	CONVERGES WITH (6)
parietal cortex	18	8	CONVERGES WITH (6)

**Table 15:** Structural characteristics of major functional knowledge clusters.

Cluster	Theme	Main Connected Diseases	Main Connected Modalities
C1	AD Multimodal Diagnostic Core	AD, MCI, dementia	PET, fMRI, CSF, EEG
C2	AD Molecular Imaging and Fluid Biomarker	AD, MCI	fMRI, CSF, tau-PET
C3	MDD Resting-State fMRI Functional Connectivity	MDD, bipolar	resting-state fMRI, EEG
C4	EEG Multi-disease Diagnostic Classification	AD, MCI, dementia	EEG, PET, fMRI
C5	EEG-fNIRS Cross-modal Fusion	MDD, chronic pain	fNIRS, fMRI, PET
C6	fMRI-PET Spatial Localization Fusion	AD, MDD, chronic pain	PET, EEG, fNIRS
C7	FDG-PET and Structural MRI Integration	FDG-PET, structural MRI, fMRI, CSF	structural MRI, fMRI, CSF

**Table 16:** Cross-modal combination characteristics.

Modality Pair	Nodes (A/B)	Direct Edges	Edge Density	Shared Neighbors	Dominant Relation Type
fMRI-PET	501/540	401	0.148%	189	DIAGNOSTICALLY SYNERGIZES
fMRI-fNIRS	501/76	107	0.281%	120	FUSES WITH, CONVERGES WITH
fNIRS-PET	76/540	116	0.283%	89	DIAGNOSTICALLY SYNERGIZES
fMRI-EEG	501/454	102	0.045%	97	FUSES WITH, SPATIALLY COMPLEMENTS
EEG-fNIRS	454/76	69	0.200%	61	DIAGNOSTICALLY SYNERGIZES
EEG-PET	454/540	42	0.017%	104	TEMPORALLY INFORMS, CONVERGES WITH

**Table 17:** Connectivity degree distribution of core disease-related nodes.

Disease	Node	Degree
MDD	major depressive disorder	198
	Major Depressive Disorder (MDD)	54
	bipolar disorder	29
	diagnostic marker MDD	13
	geriatric depression	8
AD	Alzheimer’s disease	767
	amyloid beta 42	8
	amyloid-β PET	320
	mild cognitive impairment	124
	tau-PET	156
Chronic pain	central pain processing biomarkers	3
	pain and emotional processing circuits	2
	pain severity	8
	chronic pain diagnostic marker	4
	fibromyalgia	4

**Table 18:** Clinical translation evidence.

Translation Stage	Edge Count (%)	Typical Example
Diagnostic Synergy	2881 (41.1%)	fMRI + PET combined diagnosis of AD
Treatment Guidance	226 (3.2%)	fMRI-localized TMS target
Prognosis Prediction	17 (0.2%)	MCI-to-AD conversion prediction
Technical Fusion	1464 (20.9%)	EEG-informed fMRI
Spatial Complementarity	968 (13.8%)	fMRI + fNIRS pain assessment
Temporal Complementarity	144 (2.1%)	EEG + fMRI cognitive load
Contradictory Relation	344 (4.9%)	fMRI activation vs. EEG suppression

## Data Availability

All literature records were retrieved from Web of Science, PubMed, Scopus, and PsycINFO. Restrictions apply to the availability of these third-party data. The data are available from the corresponding databases with appropriate permissions.

## References

[1] Woolgar A., Feredoes E., Assem M., Bassil Y., Bergmann T.O., Beynel L., Burke M., Cash R.F.H., Comeau R.M., Correia M.M. (2026). Consensus guidelines for the use of concurrent TMS-fMRI in cognitive and clinical neuroscience. Nat. Protoc..

[2] Kuruppu G., Wagh N., Kremen V., Varatharajah Y. (2026). EEG foundation models: A critical review of current progress and future directions. J. Neural Eng..

[3] Yang L., Wang Z. (2025). Applications and advances of combined fMRI-fNIRS techniques in brain functional research. Front. Neurol..

[4] Safarian A., Mirshahvalad S.A., Nasrollahi H., Jung T., Pirich C., Arabi H., Beheshti M. (2025). Impact of [18F]FDG PET/CT radiomics and artificial intelligence in clinical decision making in lung cancer: Its current role. Semin. Nucl. Med..

[5] Yu S., Shu C., Wang G. (2025). Abnormal static and dynamic regional homogeneity in adolescent major depressive disorder with somatic symptoms: A resting-state fMRI study. Ann. Gen. Psychiatry.

[6] Kou Y., Si Y., Liu L., Li J., Zhang Y., Li W., Zhang J., Wang C., Zhang H. (2025). Exploring the functional connectivity of resting-state EEG in adolescent major depressive disorder. J. Integr. Neurosci..

[7] Gui G., Shu C., Zong X., Liu H., Wang H., Tan H., Bai Y., Hu M., Yang M., Wang G. (2026). Altered resting-state prefrontal activity and network topology in adolescents with depression: An fNIRS study. Front. Psychiatry.

[8] Yu S., Zheng N., Ma Y., Wu H., Chen B. (2018). A novel brain decoding method: A correlation network framework for revealing brain connections. IEEE Trans. Cogn. Dev. Syst..

[9] Shen G., Horikawa T., Majima K., Kamitani Y. (2019). Deep image reconstruction from human brain activity. PLoS Comput. Biol..

[10] Wang R., Liang S., Chen Q., Huang Y., Li M., Ma Y., Zhang D., Qin K., Leung M.-F. (2025). GraphCogent: Mitigating LLMs’ Working Memory Constraints via Multi-Agent Collaboration in Complex Graph Understanding. arXiv.

[11] Blondel V.D., Guillaume J.L., Lambiotte R., Lefebvre E. (2008). Fast unfolding of communities in large networks. J. Stat. Mech. Theory Exp..

[12] Woo C.W., Chang L.J., Lindquist M.A., Wager T.D. (2017). Building Better Biomarkers: Brain Models in Translational Neuroimaging. Nat. Neurosci..

[13] Phillips M.L., Ladouceur C.D., Drevets W.C. (2008). A Neural Model of Voluntary and Automatic Emotion Regulation: Implications for Understanding the Pathophysiology and Neurodevelopment of Bipolar Disorder. Mol. Psychiatry.

[14] Drysdale A.T., Grosenick L., Downar J., Dunlop K., Mansouri F., Meng Y., Fetcho R.N., Zebley B., Oathes D.J., Etkin A. (2017). Resting-State Connectivity Biomarkers Define Neurophysiological Subtypes of Depression. Nat. Med..

[15] Jack C.R., Bennett D., Blennow K., Carrillo M.C., Dunn B., Elliott C., Haeberlein S.B., Holtzman D.M., Jagust W.J., Jessen F. (2018). NIA-AA Research Framework: Toward a Biological Definition of Alzheimer’s Disease. Alzheimer’s Dement..

[16] Kaiser R.H., Andrews-Hanna J.R., Wager T.D., Pizzagalli D.A. (2015). Large-Scale Network Dysfunction in Major Depressive Disorder: A Meta-Analysis of Resting-State Functional Connectivity. JAMA Psychiatry.

[17] Villemagne V.L., Dore V., Burnham S.C., Masters C.L., Rowe C.C. (2018). Imaging Tau and Amyloid-β Proteinopathies in Alzheimer Disease and Other Conditions. Nat. Rev. Neurol..

[18] Blennow K., Zetterberg H. (2018). Biomarkers for Alzheimer’s Disease: Current Status and Prospects for the Future. J. Intern. Med..

[19] Petersen R.C. (2004). Mild Cognitive Impairment as a Diagnostic Entity. J. Intern. Med..

[20] Dubois B., Feldman H.H., Jacova C., Hampel H., Molinuevo J.L., Blennow K., DeKosky S.T., Gauthier S., Selkoe D., Bateman R. (2014). Advancing Research Diagnostic Criteria for Alzheimer’s Disease: The IWG-2 Criteria. Lancet Neurol..

[21] Tracey I., Mantyh P.W. (2007). The Cerebral Signature for Pain Perception and Its Modulation. Neuron.

[22] Kucyi A., Davis K.D. (2015). The Dynamic Pain Connectome. Trends Neurosci..

[23] Hu L., Iannetti G.D. (2016). Painful Issues in Pain Prediction. Trends Neurosci..

[24] Davis K.D., Aghaeepour N., Ahn A.H., Angst M.S., Borsook D., Brenton A., Burczynski M.E., Crean C., Edwards R., Gaudilliere B. (2020). Discovery and Validation of Biomarkers to Aid the Development of Safe and Effective Pain Therapeutics: Challenges and Opportunities. Nat. Rev. Neurol..

[25] Wager T.D., Atlas L.Y., Lindquist M.A., Roy M., Woo C.W., Kross E. (2013). An fMRI-Based Neurologic Signature of Physical Pain. N. Engl. J. Med..

[26] U.S. Food and Drug Administration (2025). FDA Clears First Blood Test Used in Diagnosing Alzheimer’s Disease. https://www.fda.gov/news-events/press-announcements/fda-clears-first-blood-test-used-diagnosing-alzheimers-disease.

[27] Palmqvist S., Whitson H.E., Allen L.A., Suarez-Calvet M., Galasko D., Karikari T.K., Okrahvi H.R., Paczynski M., Schindler S.E., Teunissen C.E. (2025). Alzheimer’s Association Clinical Practice Guideline on the Use of Blood-Based Biomarkers in the Diagnostic Workup of Suspected Alzheimer’s Disease within Specialized Care Settings. Alzheimer’s Dement..

[28] Ang S.-H., Ho R.C., McIntyre R.S., Zhang Z., Chang S.-K., Teopiz K.M., Ho C.S.H. (2025). The Clinical Utility of Biomarkers in Diagnosing Major Depressive Disorder in Adults: A Systematic Review of Literature from 2013 to 2023. Psychiatry Investig..

[29] Mackey S., Greely H.T., Martucci K.T. (2019). Neuroimaging-Based Pain Biomarkers: Definitions, Clinical and Research Applications, and Evaluation Frameworks to Achieve Personalized Pain Medicine. Pain Rep..

[30] Davis K.D., Flor H., Greely H.T., Iannetti G.D., Mackey S., Ploner M., Pustilnik A., Tracey I., Treede R.-D., Wager T.D. (2017). Brain Imaging Tests for Chronic Pain: Medical, Legal and Ethical Issues and Recommendations. Nat. Rev. Neurol..

[31] Perluigi M., Di Domenico F., Butterfield D.A. (2024). Oxidative damage in neurodegeneration: Roles in the pathogenesis and progression of Alzheimer disease. Physiol. Rev..

[32] Ashton N.J., Janelidze S., Mattsson-Carlgren N., Binette A.P., Strandberg O., Brum W.S., Karikari T.K., González-Ortiz F., di Molfetta G., Meda F.J. (2022). Differential Roles of Aβ42/40, p-Tau231 and p-Tau217 for Alzheimer’s Trial Selection and Disease Monitoring. Nat. Med..

[33] Wang Y., Gao Y., Tang S., Lu L., Zhang L., Bu X., Li H., Hu X., Hu X., Jiang P. (2020). Large-Scale Network Dysfunction in the Acute State Compared to the Remitted State of Bipolar Disorder: A Meta-Analysis of Resting-State Functional Connectivity. EBioMedicine.

[34] Mulders P.C., van Eijndhoven P.F., Schene A.H., Beckmann C.F., Tendolkar I. (2015). Resting-State Functional Connectivity in Major Depressive Disorder: A Review. Neurosci. Biobehav. Rev..

[35] Bush G., Luu P., Posner M.I. (2000). Cognitive and Emotional Influences in Anterior Cingulate Cortex. Trends Cogn. Sci..

[36] Miller E.K., Cohen J.D. (2001). An Integrative Theory of Prefrontal Cortex Function. Annu. Rev. Neurosci..

[37] Molinuevo J.L., Ayton S., Batrla R., Bednar M.M., Blennow K. (2018). Current State of Alzheimer’s Fluid Biomarkers. Acta Neuropathol..

[38] Vieira S., Pinaya W.H.L., Mechelli A. (2017). Using deep learning to investigate the neuroimaging correlates of psychiatric and neurological disorders: Methods and applications. Neurosci. Biobehav. Rev..

[39] Van Essen D.C., Smith S.M., Barch D.M., Behrens T.E., Yacoub E., Ugurbil K. (2013). The WU-Minn Human Connectome Project: An Overview. NeuroImage.

[40] Laird A.R., Lancaster J.L., Fox P.T. (2005). BrainMap: The Social Evolution of a Human Brain Mapping Database. Neuroinformatics.

[41] Yarkoni T., Poldrack R.A., Nichols T.E., Van Essen D.C., Wager T.D. (2011). Large-scale automated synthesis of human functional neuroimaging data. Nat. Methods.

[42] Peng C., Yang X., Chen A., Smith K.E., PourNejatian N., Costa A.B., Martin C., Flores M.G., Zhang Y., Magoc T. (2023). A Study of Generative Large Language Model for Medical Research and Healthcare. npj Digit. Med..

[43] Hogan A., Blomqvist E., Cochez M., D’Amato C., De Melo G., Gutierrez C., Kirrane S., Labra Gayo J.E., Navigli R., Neumaier S. (2021). Knowledge graphs. ACM Comput. Surv..

[44] Calhoun V.D., Sui J. (2016). Multimodal Fusion of Brain Imaging Data: A Key to Finding the Missing Link(s) in Complex Mental Illness. Biol. Psychiatry Cogn. Neurosci. Neuroimaging.

[45] Insel T., Cuthbert B., Garvey M., Heinssen R., Pine D.S., Quinn K., Sanislow C., Wang P. (2010). Research Domain Criteria (RDoC): Toward a New Classification Framework for Research on Mental Disorders. Am. J. Psychiatry.

[46] Goodkind M., Eickhoff S.B., Oathes D.J., Jiang Y., Chang A., Jones-Hagata L.B., Ortega B.N., Zaiko Y.V., Roach E.L., Korgaonkar M.S. (2015). Identification of a Common Neurobiological Substrate for Mental Illness. JAMA Psychiatry.

[47] Pinti P., Tachtsidis I., Hamilton A., Hirsch J., Aichelburg C., Gilbert S., Burgess P.W. (2020). The Present and Future Use of Functional Near-Infrared Spectroscopy (fNIRS) for Cognitive Neuroscience. Ann. N. Y. Acad. Sci..

[48] Fox M.D., Buckner R.L., White M.P., Greicius M.D., Pascual-Leone A. (2012). Efficacy of Transcranial Magnetic Stimulation Targets for Depression Is Related to Intrinsic Functional Connectivity with the Subgenual Cingulate. Biol. Psychiatry.

[49] Cole E.J., Phillips A.L., Bentzley B.S., Stimpson K.H., Nejad B.S.R., Barmak F., Williams N.R. (2022). Stanford Neuromodulation Therapy (SNT): A Double-Blind Randomized Controlled Trial. Am. J. Psychiatry.

[50] van Dyck C.H., Swanson C.J., Aisen P., Bateman R.J., Chen C., Gee M., Kanekiyo M., Li D., Reyderman L., Cohen S. (2023). Lecanemab in Early Alzheimer’s Disease. N. Engl. J. Med..

